# The Cost-Effectiveness of Hyperlipidemia Medication in Low- and Middle-Income Countries: A Review

**DOI:** 10.5334/gh.1097

**Published:** 2022-03-04

**Authors:** Muhammad Jami Husain, Garrison Spencer, Rachel Nugent, Deliana Kostova, Patricia Richter

**Affiliations:** 1 Division of Global Health Protection, Centers for Disease Control and Prevention, Atlanta, US; 2 Center for Global Noncommunicable Diseases, RTI International, Seattle, Washington, US

**Keywords:** Hyperlipidemia, Cost-effectiveness, Low and Middle Income Countries

## Abstract

Hyperlipidemia is a risk factor for cardiovascular disease – the leading cause of death globally. Increased understanding of the cost-effectiveness of hyperlipidemia treatment in low- and middle-income countries can guide approaches to hyperlipidemia management in resource-limited environments. We conducted a systematic review of the evidence on the cost-effectiveness of hyperlipidemia medication treatment in low- and middle-income countries using studies published between January 2010 and April 2020. We abstracted study details, including study design, treatment setting, intervention type, health metrics, costs standardized to constant 2019 US dollars, and cost-effectiveness measures including average and incremental cost-effectiveness ratios. Comparisons across studies suggested that treatment via polypill is generally more cost-effective than statin-only therapy, and that primary prevention is more cost-effective than secondary prevention. Treating hyperlipidemia at a threshold of 5.7 mmol/l comes at a higher cost per disability-adjusted life-years averted than at a threshold of 6.2 mmol/l. Most pharmacological treatment strategies for hyperlipidemia were found to be cost-effective in most of the examined low- and middle-income countries.

## Introduction

Hyperlipidemia is a major risk factor for cardiovascular disease (CVD) – the leading cause of morbidity and mortality globally [1]. The number and proportion of CVD-attributable deaths in low- and middle-income countries (LMICs) have been steadily increasing over the past 30 years [2]. The rising burden of CVD in LMICs has been linked to increased levels of risk factors such as hypertension, diabetes, tobacco use, obesity, physical inactivity, and hyperlipidemia [2].

Hyperlipidemia encompasses lipoprotein disorders, including elevated levels of total cholesterol, low-density lipoprotein (LDL) cholesterol, non-high-density lipoprotein (non-HDL) cholesterol, and triglycerides, as well as low HDL cholesterol. The World Health Organization (WHO) defines elevated cholesterol as total cholesterol greater than 5.0 mmol/L (190 mg/dl) and very high cholesterol as total cholesterol greater than 6.2 mmol/L (240 mg/dl) [3]. Most hyperlipidemia patients have a primary lipid disorder, while secondary hyperlipidemia can arise from other conditions such as diabetes mellitus, excessive alcohol intake, kidney disease (e.g., nephrotic syndrome or chronic renal failure), liver disease (e.g., cholestasis), hypothyroidism, obesity, and some medications (e.g., thiazide diuretics, beta blockers, atypical antipsychotics, and HIV medications) [4]. Familial hyperlipidemia can occur from mutations of critical lipid metabolism genes and is associated with very high levels of LDL-C (>= 190 mg/dl), strong family history of premature CVD, and the occurrence of cardiovascular events at younger ages [5].

Cholesterol screening aims to reduce downstream CVD morbidity. Hyperlipidemia is a common, asymptomatic, pre-clinical condition that is modifiable through behavioral and pharmacological interventions. Treatment of hyperlipidemia can enhance primary and secondary prevention of CVD events [6,7,8,9,10,11] and can be cost-effective for patients at high risk of CVD [12,13,14,15]. Treatment decisions are guided by overall CVD risk estimation, existing guidelines, and in some cases, absolute cholesterol levels. All patients with hyperlipidemia should receive counseling on lifestyle interventions including diets rich in fruits and vegetables and adequate physical activity [16,17,18,19,20]. Where lifestyle modification is not sufficient, several classes of medications can be used. HMG-CoA reductase inhibitors, better known as statins, make up the vast majority of lipid lowering medications currently in use [21,22]. Other medications used to treat hyperlipidemia include ezetimibe, fibrates, bile acid sequestrants, niacin, proprotein convertase subtilisin/kexin type 9 (PCSK9) inhibitors, and monoclonal antibodies. However, statins are the only lipid lowering medication on the World Health Organization (WHO) Model List of Essential Medicines [21].

While the rate of screening and treatment for hyperlipidemia is high in high-income countries (HICs), it remains low in LMICs. Uptake of hyperlipidemia treatment is often cost-dependent, but its cost-effectiveness in LMICs is not well understood. This literature review aims to collect and summarize the existing evidence on the cost-effectiveness of hyperlipidemia treatment in LMICs. It aims to identify types of medications and treatment strategies associated with higher cost-effectiveness, helping to inform decisions regarding approaches to lipid control in LMICs.

## Methods

### Eligibility criteria

The review included published studies of patients of any age treated with any lipid-altering medication or were subject to behavioral interventions for addressing hyperlipidemia. Treated subjects were included regardless of the types of hyperlipidemia diagnosis, unless treatment occurred after surgery or for an acute condition, such as heart procedures or subarachnoid hemorrhage. Lipid-altering medication interventions for secondary prevention were also included. Interventions involving a polypill (fixed-dose combination of CVD medications, including a statin) were included, while studies of herbal medicines, population-level interventions, and screening interventions were excluded. Geographic criteria were limited to countries classified as LMICs currently or within the past five years.

Original research studies using cost-effectiveness, cost-benefit, or cost-utility analysis were eligible for inclusion in this systematic review. Types of study design included randomized controlled trials (RCTs), quasi-experimental, longitudinal, cross-sectional, or observational studies. Modelled analyses, including those based on secondary data, were also included. Editorials, article or book reviews, correspondence, abstracts, poster or oral presentations, protocol or study designs, and non-systematic reviews were excluded.

### Data sources and search strategy

We searched several medical and economic literature databases from January 1, 2010 to April 2020, including PubMed, EMBASE, EconLit, Cochrane Database of Systematic Reviews, UK National Institute for Health and Care Excellence (NICE), Tufts Medical Center Cost-Effectiveness Analysis Registry, Disease Control Priorities 3^rd^ Edition (DCP3), and the Thailand Health Intervention and Technology Assessment Program (HITAP). The PubMed search strategy, which was adapted for each of the other sources, is provided in Supplementary Table 1. Two independent reviewers screened the titles and abstracts of the initially identified studies to determine if they satisfied the selection criteria. An inclusion/exclusion guide was created to assist in reviewing the abstract. Conflicts in determining abstract eligibility were resolved by consensus, with referral to a third reviewer, as necessary.

### Data abstraction

We used an Excel template to summarize study details, including author information, article title, publication year, country, World Bank country income classification, World Bank regional classification, study design, study population (number size and description), intervention (type, description, and duration), comparator (including the existence of a null/status-quo scenario), outcomes reported, cost-effectiveness measures reported, analysis perspective, currency units, currency year, discount rate, and whether a sensitivity analysis was performed. Abstracted information on costs included data availability on direct and indirect costs, capital costs, and listed the types of costs included in the cost-effectiveness analysis.

Data on cost-effectiveness measures were abstracted for quality-adjusted life-years (QALYs) gained, disability-adjusted life-years (DALYs) averted, and other cost-effectiveness measures. Lastly, a worksheet each was created to capture study costs and effects. All monetary figures were standardized by converting local currency units to US dollars and transforming them to constant 2019 US dollars using the historical consumer price index (CPI) for urban consumers provided by the Bureau of Labor Statistics. Several studies reported costs in international dollars; if the local currency to international dollar exchange rate used was provided in the paper, costs were converted to local currencies and then converted to 2019 US dollars using the process described above. If the local currency to international dollar exchange rate was not provided in the paper, the relevant exchange rate from the World Bank World Development Indicators Database was used.

## Results

### Literature search

**Figure 1:**
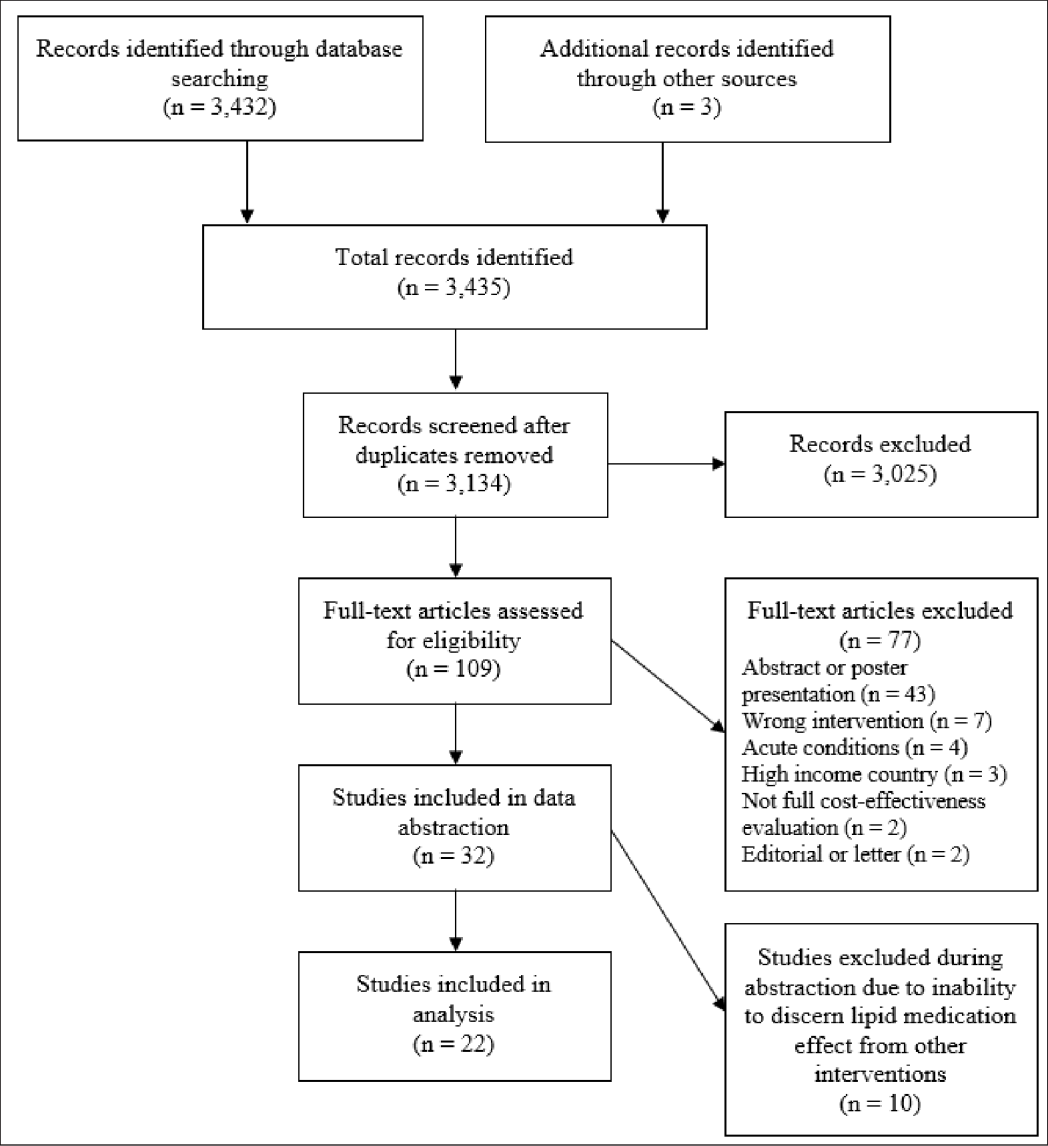
PRISMA flow diagram.

The initial database search yielded 3,432 records, and three additional studies were obtained from searching bibliographies (***Figure 1***). After removing duplicates, 3,134 studies remained for abstract review. One hundred nine articles were identified as potentially eligible for inclusion in the analysis and were read by two independent reviewers. Of these, 32 were studies qualified for data abstraction. Following data abstraction, 10 studies were excluded because the cost-effectiveness of hyperlipidemia medications was not provided or was not disaggregated from other interventions. For example, Basu, Bendavid, and Sood [23] model the cost-effectiveness of primary prevention using pharmacological treatment for hypertension and high cholesterol in India. However, the cost-effectiveness measures are not disaggregated by the individual interventions and so the cost-effectiveness of pharmacological treatment of high cholesterol alone cannot be obtained. Because these studies do include pharmacological interventions for hyperlipidemia and may be of interest to the reader, a list of the articles is included in Supplementary Table 2.

### Characteristics of included studies

Over half of the included studies (n = 13) come from upper middle-income countries [24,25,26,27,28,29,30,31,32,33,34,35,36], four are from lower middle-income countries [37,38,39,40], two are from low-income countries [41,42], and three contain countries of multiple income levels or conduct analysis at a regional level [43,44,45]. Studies come from every region except North America, with seven from Latin America and Caribbean [24,25,28,29,31,35,44], seven from East Asia and the Pacific [26,27,32,33,34,37], two from South Asia [38,40], two from Sub-Saharan Africa [41,42], one from Europe [30], one from Middle East [36], and two with countries from multiple regions [43,45]. These studies produce results for 14 individual countries: Argentina [25,31], Brazil [28,29], Bulgaria [30], Colombia [28], China [32,34,45], Ethiopia [42], India [38,40,45], Iran [36], Mexico [24,35,45], Ghana [45], Philippines [39], South Africa [45], Tanzania [41], Thailand [26,27,33], and Vietnam [37]. Supplementary Table 3 summarizes the study characteristics with details about intervention type, treatment setting, outcomes reported, study findings, and other methodological attributes.

Seven studies reported DALYs averted (shown in Supplementary Table 4), nine reported QALYs gained (Supplementary Table 5), four reported CVD events averted (Supplementary Table 6), four reported life-years gained (Supplementary Table 7), and two measured reductions in lipid levels (Supplementary Table 8). Depending on the study, the interventions were compared to either a ‘no intervention’ (null scenario) or a ‘status quo’ (standard care) scenario, and in some cases followed by incremental analysis between mutually exclusive interventions. We report the average cost effectiveness ratios (ACERs) in 2019 USD, which are defined as the ratio of intervention cost per health effect. The studies that report incremental cost-effectiveness ratios (ICERs) estimate the ICERs as the ratio of the incremental cost to incremental effects for moving from one intervention to the next more effective intervention, starting from the null scenario (e.g., often this is the standard or existing care). Interventions with higher ICERs (i.e., more costly, and less effective) than their more effective comparator are designated as dominated. We report ACERs of respective interventions (i.e., statins or polypills in primary or secondary preventive care settings) and ICERs compared to standard care (as defined in respective studies) in dollars per health outcome of interest (e.g., QALY gained, DALY averted, or CVD events averted) in 2019 USD in the supplementary tables. The ACERs and ICERs were assessed as a percentage of country GDP per capita in 2019 USD.

In most of the studies (n = 17), statins were the only lipid-lowering drug included; however, other lipid-lowering drugs were represented. Three studies include ezetimibe [24,33,34], two studies include a PCSK9 inhibitor [30,33], and one includes fibric acid [39]. Six studies included a polypill (a fixed-dose combination pill usually containing a statin, three types of blood pressure medication, and aspirin) [25,27,38,40,44,45]. Ten studies considered both primary and secondary prevention [24,26,28,29,30,31,35,38,42,43], seven involved only primary prevention [25,27,32,36,37,41,44], and the remaining five involved only secondary prevention [33,34,39,40,45]. One study included only patients with heterozygous familial hyperlipidemia [30] and one included only patients with newly diagnosed type 2 diabetes mellitus [32]. While some studies did include lifestyle modification, this was delivered by a physician at the same time as hyperlipidemia medication, often as described by the WHO-CHOICE CVD intervention number seven, ‘Cholesterol-lowering drug treatment (statins) and education (ED) on lifestyle modification including dietary advice, delivered by physicians to individuals whose serum cholesterol concentration (CHOL) exceeds 220 mg/dl (5.7 mmol/l)’ [46].

### Cost-effectiveness of treatment with statins and polypills

**Figure 2:**
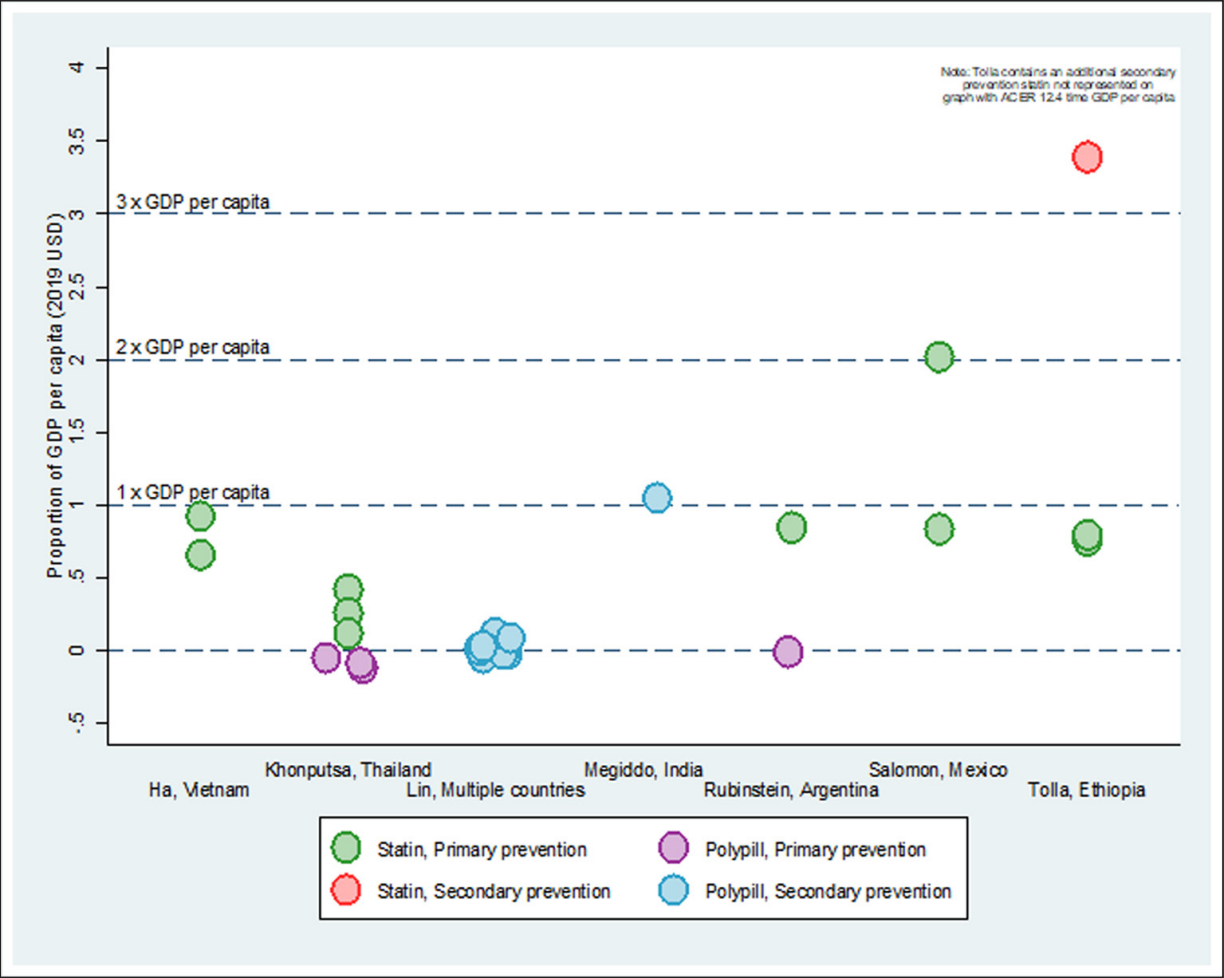
**Average cost-effectiveness of treating cholesterol per DALY averted relative to GDP per capita.** *Note*: The overlapping circles (i.e., with similar or very close ACER values) are presented as jittered around the respective values for better visualization of the number of interventions assessed. See Supplementary Table 4 for study- and intervention-specific ACERs, treatment description, and intervention sub-groups.

A rule of thumb for assessing cost-effectiveness is the relative size of the cost-effectiveness indicator to country GDP per capita, where an intervention is considered cost-effective if it is below three times the GDP per capita threshold and very cost-effective if below the GDP per capita threshold [47]. Cross-study comparison of cost per DALY averted relative to GDP per capita shows that statin monotherapy and polypill with statins are cost-effective for hyperlipidemia treatment (***Figure 2***). Further, statin monotherapy and polypill are both cost effective for primary and secondary prevention. Most of the ACER values from the seven studies in ***Figure 2*** show costs per DALY that are less than 1 percent of GDP per capita, which makes the interventions very cost-effective. An exception is found in Ethiopia, where secondary prevention of stroke and ischemic heart disease reported costs per DALY averted over 3 and 12 times greater than GDP per capita, respectively [42]. Primary prevention, however, was found to be very cost-effective in Ethiopia, with costs of around 0.8 times GDP per capita [42].

Polypill was used for both primary and secondary prevention. In Thailand, Khonputsa et al. found that a polypill would be cost-effective even for those with a 10-year risk of 5 to 9.9 percent, with an average cost-effectiveness of US $-367 per DALY averted (includes cost offsets of medical expenditures averted) [27]. In India, Megiddo et al. estimates the average cost-effectiveness of a polypill for secondary prevention of myocardial infarction of US $2,063 per DALY averted (slightly more than GDP per capita of US $2,046) [40]. Lin et al. estimated the effect of polypill treatment among those with atherosclerotic CVD in five countries, including India, and finds the cost per DALY averted to be lowest there (US $18), followed by Mexico (US $87), South Africa (US $103), China (US $130), and Nigeria (US $143) [45]. For secondary prevention in rural India, Wood et al. estimated an average cost-effectiveness of a polypill of US $5,457 per CVD event averted for individuals with CVD and US $6,246 for those with CVD and a >20 percent risk of experiencing a coronary heart disease event in the next ten years (Supplementary Table 6) [38].

**Figure 3:**
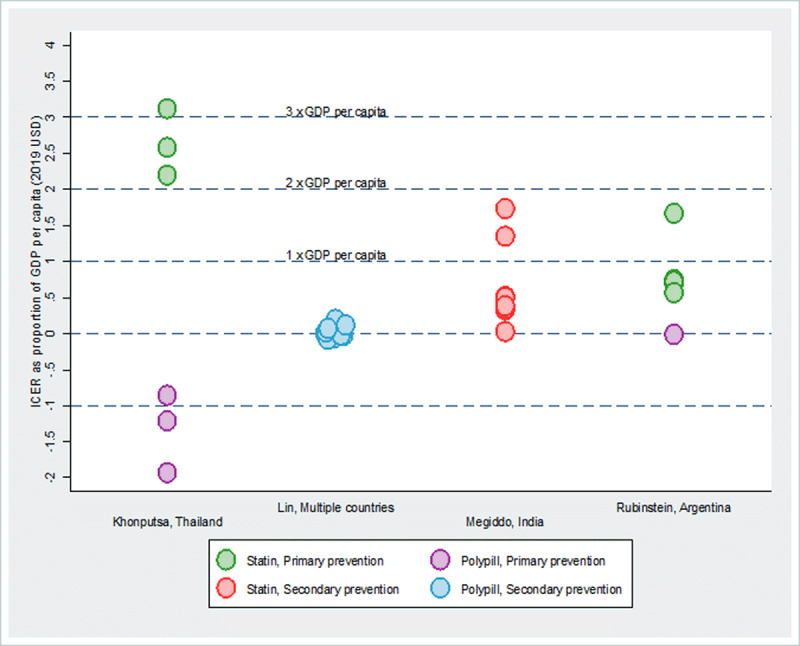
**Incremental cost-effectiveness of treating cholesterol per DALY averted relative to GDP per capita** *Note*: The overlapping circles (i.e., with similar or very close ACER values) are presented as jittered around the respective values for better visualization of the number of interventions assessed. See Supplementary Table 4 for study- and intervention-specific ICERs.

The studies that reported ICERs compared the incremental costs and effects for many alternative interventions, often as modelled scenarios. The ICERs need to be interpreted cautiously and only in terms of the respective definitions of the comparators. Khonputsa et al. reported several ICERs for inclusion of statins or polypills in the treatment protocol and compared those with current practices and found polypills to be the dominant (cost saving) strategies (***Figure 3***) [27]. Lin et al., in their multi-country studies (five countries; public and private sectors) estimated the ICERs for using polypills compared to the current practice entailing patients received aspirin 75 mg, lisinopril 10 mg, atenolol 50 mg, and simvastatin 40 mg in separate individual pills at prescription levels and used for secondary prevention of CVD. As seen in ***Figure 3***, the ICERs ranged from 0.5 percent to 13.4 percent of respective GDP per capita [45]. Rubenstein et al. modelled the ICERs of several interventions compared to no intervention measured as cost per averted DALY; the ICERs were in the range of –1.3 percent (cost-saving) to 167 percent of GDP per capita [25].

**Figure 4:**
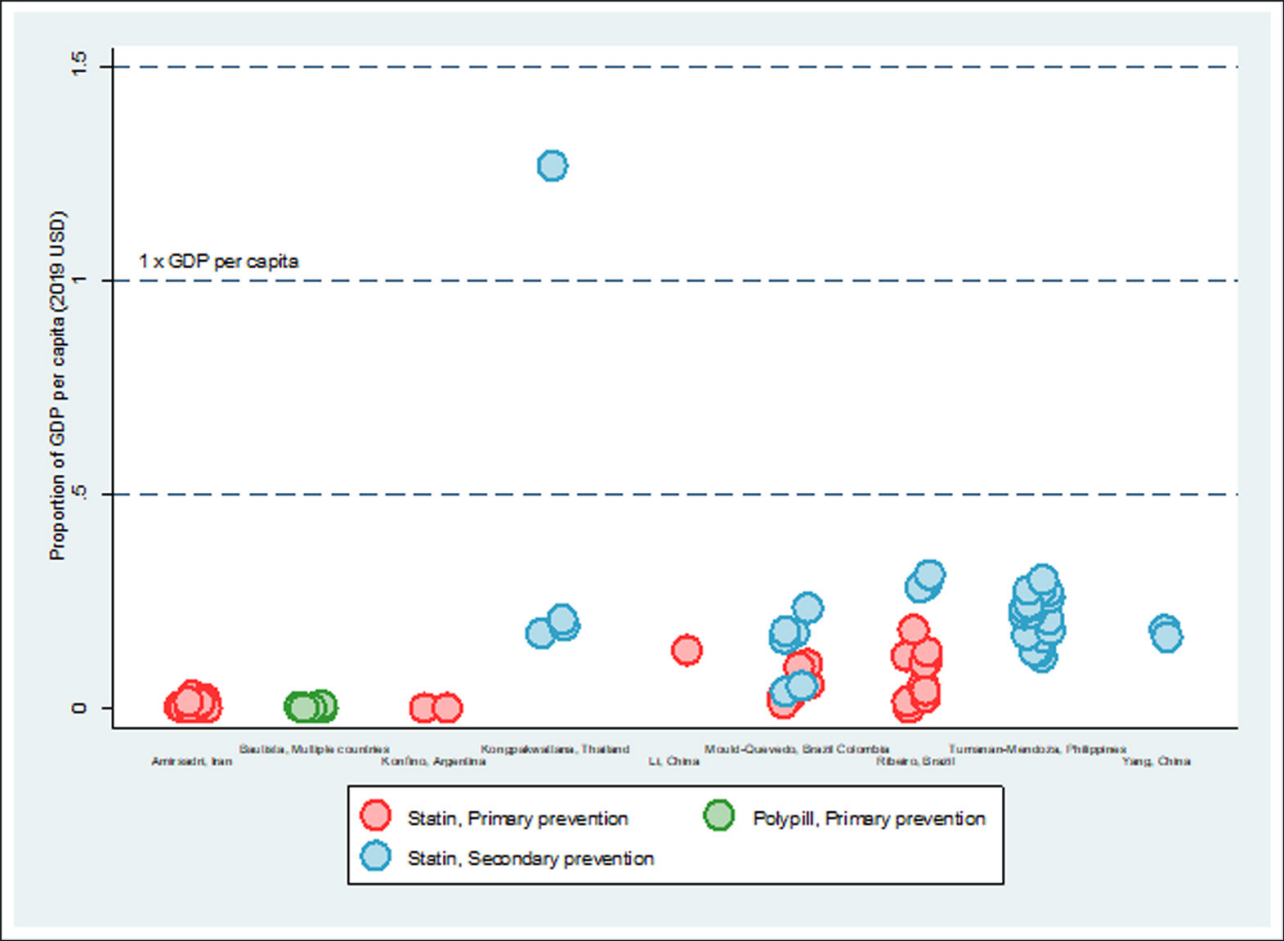
**Average cost-effectiveness of treating cholesterol per QALY gained and relative to 1x GDP per capita, 2019 USD** *Note*: The overlapping circles (i.e., with similar or very close ACER values) are presented as jittered around the respective values for better visualization of the number of interventions assessed. See Supplementary Table 5 for study- and intervention-specific ACERs.

Using nine studies that report cost per QALY gained and distinguished between primary and secondary prevention, cross-study analysis shows that the cost per QALY gained is consistently lower for primary prevention compared to secondary prevention (***Figure 4***). For primary prevention, Bautista et al. modeled the effects of providing a hypothetical polypill in eight Latin American countries (not disaggregated). The intervention is most cost-effective for women with a 10-year CVD risk of 15 percent or more (average cost-effectiveness of USD 45 per QALY gained) and men age 55 and older (average cost-effectiveness of USD 40 per QALY gained) [44]. The primary prevention interventions do not exceed 0.2 times GDP per capita, while most secondary prevention strategies fall within 0.2 to 0.3 times GDP per capita, with Kongpakwattana et al. showing the only intervention that exceeds 1 times GDP per capita (a PCSK9 inhibitor) [33].

### Average cost-effectiveness of treating cholesterol at different thresholds

**Figure 5:**
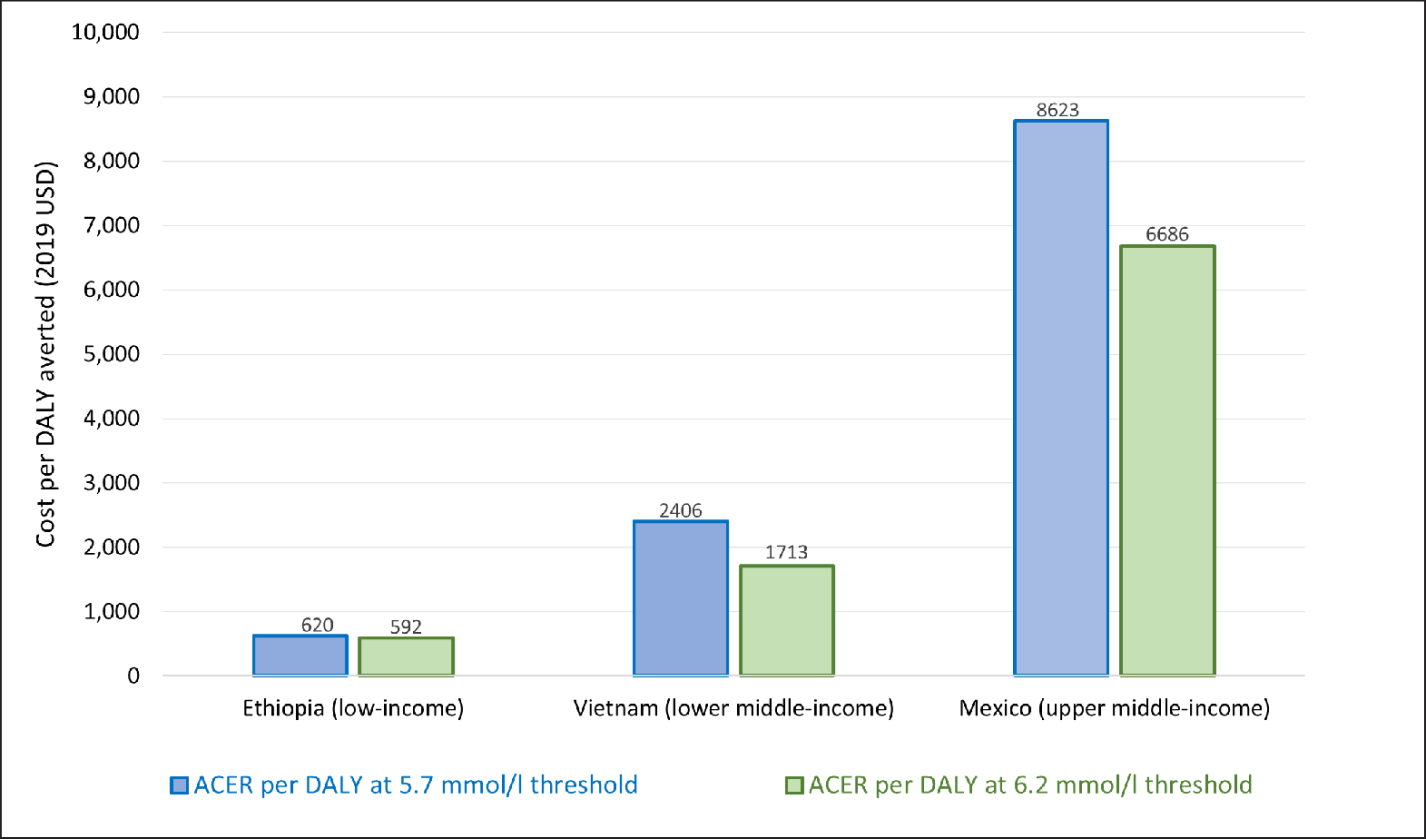
Average cost-effectiveness ratio (ACER) of treating cholesterol, 2019 USD.

Of the 13 studies that include only primary prevention or disaggregate primary prevention results from secondary prevention results, four compare the average cost effectiveness ratio (ACER) of treating patients with cholesterol at the 5.7 mmol/l and 6.2 mmol/l levels. Average cost-effectiveness ratio is the ratio of total costs to total effect, that is, akin to the incremental cost-effectiveness ratio compared to no intervention scenario (i.e., absence of care). As expected, the average cost per DALY averted is higher using the lower threshold in all studies. Using the GDP per capita threshold for cost-effectiveness [47], the three studies conducted in specific countries (excluding Ortegon et al. [43] who conducted a regional analysis and calculated cost-effectiveness from a population level) show that these interventions are all very cost-effective (i.e., less than one times GDP per capita). In Ethiopia (low-income), Tolla et al. report the average cost-effectiveness of treating at a threshold of 5.7 mmol/l is US $620 per DALY averted and treating at the threshold of 6.2 mmol/l is US $592 per DALY averted [42]; in Vietnam (lower middle-income) Ha et al. report the cost-effectiveness of the 5.7 mmol/l threshold as US $2,406 per DALY averted and the 6.2 mmol/l threshold as US $1,713 [37]; and in Mexico (upper-middle-income) Salomon et al. reports the cost-effectiveness of the 5.7 mmol/l threshold as US $8,623 per DALY averted and the 6.2 mmol/l threshold as US $6,686 per DALY averted [35] (***Figure 5***). While these represent a great range in costs, they all fall below one times GDP per capita of the respective country. Ortegon et al. report the average cost per DALY averted at a regional level and is also not easily compared due to calculating costs from a population level, resulting in lower costs per outcome. Ortegon et al. reports the average cost-effectiveness of treating at a 5.7 mmol/l threshold as US $423 and US $361 per DALY averted for sub-Saharan Africa and South Asia, respectively, and the average cost-effectiveness of treating at a 6.2 mmol/l threshold as US $335 and US $286 per DALY averted for sub-Saharan Africa and South Asia, respectively [43].

### Non-statin lipid-lowering drugs

In Bulgaria, Borissov examines the effect of adding PCSK9 inhibitor treatment to statin treatment for patients with heterozygous familial hyperlipidemia (with or without existing CVD) [30]. The incremental cost per life-year gained of adding the PCSK9 inhibitor to statin therapy is US $77,952. In Thailand, Kongpakwattana analyzes the effect of adding PSCK9 inhibitor for secondary prevention and finds that it is not cost effective at a cost of US $1.8 million per CVD event avoided and an incremental cost per QALY gained of US $547,316, compared to statin therapy alone [33]. Kongpakwattana et al. also found that ezetimibe was not cost-effective for secondary prevention, with an incremental cost per QALY gained of US $27,354 [33]. Also, for patients needing secondary prevention, in China, Yang compared a high-dose rosuvastatin to a moderate-dose rosuvastatin in combination with ezetimibe [34]. The addition of ezetimibe was found to be cost effective with an incremental cost per QALY gained of US $7,271. In Mexico, where Briseno compared the rosuvastatin to a fixed-dose combination of ezetimibe and simvastatin among a combined primary and secondary prevention population, the ezetimibe/simvastatin group had a lower proportion of patients reach their cholesterol target goals at a higher cost [24]. Only one study considered fibric acid. In the Philippines, Tumanan-Mendoza and Mendoza compared gemfibrozil to three different statins in a secondary prevention setting and found that the cost-effectiveness was strongly dominated by the statins [39].

## Discussion

This systematic review of the cost-effectiveness of medications for hyperlipidemia in LMICs identified 22 studies that differentiated the impact of hyperlipidemia medications from other CVD medications or examined the impact of polypills. Thirteen of these studies represented upper-middle-income countries, and all geographic regions were represented with at least one country.

The majority of pharmacological treatment strategies for hyperlipidemia were found to be cost-effective or very cost-effective. Treating high cholesterol at a threshold of 5.7 mmol/l comes at a higher cost per DALY averted than at a threshold of 6.2 mmol/l. Cross-study comparison shows that treatment with polypills is generally a more cost-effective strategy than statin therapy. Primary prevention tends to be more cost-effective than secondary prevention, a pattern that was especially apparent for studies reporting the cost per QALY gained. This reflects the potential of primary prevention to generate a larger aggregate health benefit for lower unit delivery costs, as secondary prevention requires more frequent follow-up at hospital facilities.

This review is subject to a number of limitations. Because it is based on a diverse set of interventions and geographies, the comparability between estimates is broad. The articles in this review do not provide focused insights into the impact of access to medications or behavioral interventions such as diet and exercise [48]. The review uses the GDP threshold of cost-effectiveness. This approach has limitations in terms of reflecting affordability and feasibility; however, it remains the standard approach for conducting cross-country comparisons. The studies included in the review are conducted mostly from the health-systems perspective rather than societal perspective, providing a narrower view of overall impact. Further studies on the accessibility and affordability of different hyperlipidemia interventions, conducted from different perspectives (e.g., health systems vis-à-vis societal perspective) are essential.

The rising burden of CVD and the limited availability of treatment in LMICs motivates the analysis of cost-effectiveness of hyperlipidemia treatment. Polypill treatment approaches have warranted special consideration as an effective strategy for CVD prevention in LMICs [49,50,51]. The WHO Expert Committee on the Selection and Use of Essential Medicines is reviewing an application for including a fixed dose combination therapy using aspirin, statin and antihypertensive in the model list of essential medicines [52]. In lower-resource environments, polypill use may improve access and affordability of pharmaceutical CVD prevention, with relatively higher cost-effectiveness than individual medication use.

## Conclusion

Comparisons across studies suggested that treatment via polypill is generally more cost-effective than statin-only therapy, and that primary prevention is more cost-effective than secondary prevention. Treating hyperlipidemia at a threshold of 5.7 mmol/l comes at a higher cost per disability-adjusted life-years averted than at a threshold of 6.2 mmol/l. Pharmacological treatment strategies for hyperlipidemia had varying levels of cost-effectiveness across countries, formulations and treatment thresholds, but most were found to be cost-effective in most of the examined LMICs.

**Disclaimer:** The findings and conclusions in this report are those of the authors and do not necessarily represent the official position of the Centers for Disease Control and Prevention.

## Additonal File

The additonal file for this article can be found as follows:

10.5334/gh.1097.s1 Supplementary Tables. Tables 1 to 9.
