# Supplementary material for: The Cost-Effectiveness of Hyperlipidemia Medication in Low- and Middle-Income Countries: A Review
Source: Glob Heart. 2022 Mar 4;17(1):18. doi: 10.5334/gh.1097 (PMC8896253; doi:10.5334/gh.1097)
Supplement: Supplementary Tables. — Tables 1 to 9. [file gh-17-1-1097-s1.pdf]

Supplementary Table 1: PubMed Search Strategy Conducted on April 15, 2020

Search	Query
1	(Africa OR Asia OR Caribbean OR "West Indies" OR "South America" OR "Latin America" OR "Central America" OR "Middle East" OR "Eastern Europe" OR Oceania)
2	(Abkhazia OR Afghanistan OR Albania OR Algeria OR Angola OR Antigua OR Barbuda OR Argentina OR Armenia OR Armenian OR Artsakh OR Aruba OR Azerbaijan OR Bahamas OR Bangladesh OR Barbados OR Benin OR Byelarus OR Byelorussian OR Belarus OR Belorussian OR Belorussia OR Belize OR Bermuda OR Bhutan OR Bolivia OR Borneo OR Bosnia OR Herzegovina OR Hercegovina OR Botswana OR Brasil OR Brazil OR Bulgaria OR "Burkina Faso" OR "Burkina Fasso" OR "Upper Volta" OR Burundi OR Urundi OR Cambodia OR "Khmer Republic" OR Kampuchea OR Cameroon OR Cameroons OR Cameron OR Camerons OR "Cape Verde" OR "Cabo Verde" OR "Central African Republic" OR Chad OR Tchad OR Chile OR China OR Colombia OR Comoros OR "Comoro Islands" OR Comores OR Congo OR DRC OR "Congo-Brazzaville" OR "Congo-Kinshasa" OR Zaire OR "Cote d'Ivoire" OR "Ivory Coast" OR Croatia OR Cuba)
3	(Djibouti OR "French Somaliland" OR Dominica OR "Dominican Republic" OR "East Timor" OR "East Timur" OR "Timor Leste" OR "Timor-Leste" OR Ecuador OR Egypt OR "United Arab Republic" OR "El Salvador" OR Eritrea OR Ethiopia OR Fiji OR Gabon OR "Gabonese Republic" OR Gambia OR Gaza OR Georgia OR Georgian OR Ghana OR "Gold Coast" OR Grenada OR Guatemala OR Guinea OR Guiana OR Guyana OR Haiti OR Honduras OR India OR Maldives OR Indonesia OR Iran OR Iraq OR Jamaica OR Jordan OR Kazakhstan OR Kazakh OR Kenya OR Kiribati OR Korea OR DPRK OR Kosovo OR Kyrgyzstan OR Kirghizia OR "Kyrgyz Republic" OR Kirghiz OR Kirgizstan OR "Lao PDR" OR Laos OR Lebanon OR Lesotho OR Basutoland OR Liberia OR Libya)
4	(Macedonia OR FYROM OR Macao OR Madagascar OR "Malagasy Republic" OR Malaysia OR Malaya OR Malay OR Sabah OR Sarawak OR Malawi OR Nyasaland OR Mali OR "Marshall Islands" OR Mauritania OR Mauritius OR "Agalega Islands" OR Mexico OR Micronesia OR Moldova OR Moldovia OR Moldovian OR Mongolia OR Montenegro OR Morocco OR Ifni OR Mozambique OR Myanmar OR Myanma OR Burma OR Namibia OR Nauru OR Nepal OR Nicaragua OR Niger OR Nigeria OR Niue OR Pakistan OR Palau OR Palestine OR Panama OR Paraguay OR Peru OR Philippines OR Philipines OR Phillipines OR Phillippines OR Polynesia)
5	(Romania OR Rumania OR Roumania OR Russia OR Russian OR Rwanda OR Ruanda OR "Saint Kitts" OR "St Kitts" OR Nevis OR "Saint Lucia" OR "St Lucia" OR "Saint Vincent" OR "St Vincent" OR Grenadines OR Samoa OR "Samoan Islands" OR "Navigator Island" OR "Navigator Islands" OR "Sao Tome" OR Principe OR Senegal OR Serbia OR Montenegro OR "Sierra Leone" OR "Sri Lanka" OR Ceylon OR "Solomon Islands" OR Somalia OR Somaliland OR "South Africa" OR "South Ossetia" OR Sudan OR Suriname OR Surinam OR Swaziland OR Eswatini OR Syria OR Tajikistan OR Tadzhikistan OR Tadjikistan OR Tadzhik OR Tanzania OR Thailand OR Tibet OR Togo OR "Togolese Republic" OR Tokelau OR Tonga OR Transnistria OR Trinidad OR Tobago OR Tunisia OR Turkey OR Turkmenistan OR Turkmen OR Tuvalu OR Uganda OR Ukraine OR Uruguay OR USSR OR "Soviet Union" OR "Union of Soviet Socialist Republics" OR Uzbekistan OR Uzbek OR Vanuatu OR "New Hebrides" OR Venezuela OR Vietnam OR "Viet Nam" OR "Mekong valley" OR "Mekong delta" OR "Western Sahara" OR Sahrawi OR "West Bank" OR Yemen OR Yugoslavia OR Zambia OR Zimbabwe OR Zanzibar OR Rhodesia)
6	("Developing Countries"[Mesh] OR LMIC OR LMICS OR LEDC OR "less developed country" OR "least developed countries" OR "newly industrialized countries" OR "emerging markets" OR "frontier markets" OR "poor countries" OR "poor country" OR "less economically developed country" OR "underdeveloped country" OR "low-income country" OR "low-income countries" OR "middle-income country" OR "middle-income countries" OR "low- and middle-income countries" OR "developing country" OR "developing world" OR "third world" OR "less developed countries" OR "developing nations" OR "low GDP" OR "low HDI" OR "transitional economies" OR "Global South")
7	1 OR 2 OR 3 OR 4 OR 5 OR 6 OR 7
8	("Hyperlipidemias"[Mesh] OR "Dyslipidemias"[Mesh] OR cholesterol OR triglycerides OR lipid OR lipids OR "Hypercholesterolemia"[Mesh] OR "Hyperlipidemia, Familial Combined"[Mesh] OR "Hyperlipoproteinemias"[Mesh] OR "Hypertriglyceridemia"[Mesh])
9	("Hydroxymethylglutaryl-CoA Reductase Inhibitors"[Mesh] OR "Fibric Acids"[Mesh] OR "Ezetimibe"[Mesh] OR "Cholestyramine Resin"[Mesh] OR "bile acid sequestrant" OR "Proprotein Convertase 9"[Mesh] OR "Niacin"[Mesh] OR atorvastatin OR fluvastatin OR lovastatin OR pravastatin OR rosuvastatin OR simvastatin OR pitavastatin OR cerivastatin OR

	mevastatin OR statin OR statins)
**10**	8 OR 9
**11**	("Cost-effectiveness" OR "cost effectiveness" OR "cost effective" OR "cost-effective" OR "economic evaluation" OR "Costs and Cost Analysis"[Mesh] OR "Cost Sharing"[Mesh] OR "Cost Savings"[Mesh] OR "Cost of Illness"[Mesh] OR "Economics"[Mesh] OR "Cost-Benefit Analysis"[Mesh] OR "Health Care Costs"[Mesh] OR "Direct Service Costs"[Mesh] OR "Hospital Costs"[Mesh] OR "Employer Health Costs"[Mesh] OR "Drug Costs"[Mesh] OR "Health Expenditures"[Mesh] OR "Health Services Accessibility"[Mesh] OR "Public Health/economics"[Mesh] OR "Economics, Pharmaceutical"[Mesh])
**12**	7 AND 10 AND 11
**13**	12 AND ("2010/01/01"[Date - Publication] : "3000"[Date - Publication])) AND "english"[Language]) AND "humans"[Filter])

Supplementary Table 2: Excluded articles containing cholesterol medication interventions.

First Author	Article Title	Pub. Year	Country	WB Income Level	Region
**Balbay**	The impact of addressing modifiable risk factors to reduce the burden of cardiovascular disease in Turkey.	2019	Turkey	Upper middle	Europe and Central Asia
**Basu**	Health and Economic Implications of National Treatment Coverage for Cardiovascular Disease in India: Cost-Effectiveness Analysis	2015	India	Lower middle	South Asia
**Basu**	Implications of scaling up cardiovascular disease treatment in South Africa: a microsimulation and cost-effectiveness analysis	2019	South Africa	Upper middle	Sub-Saharan Africa
**Basu**	Alternative Strategies to Achieve Cardiovascular Mortality Goals in China and India: A Microsimulation of Target- Versus Risk-Based Blood Pressure Treatment.	2016	India and China	Multiple	Multiple
**De Smedt**	Cost-effectiveness of optimized adherence to prevention guidelines in European patients with coronary heart disease: Results from the EUROASPIRE IV survey.	2018	Multiple	Multiple	Multiple
**De Smedt**	Cost-effectiveness of optimizing prevention in patients with coronary heart disease: the EUROASPIRE III health economics project.	2012	Multiple	Multiple	Multiple
**Stevens**	Estimating the future burden of cardiovascular disease and the value of lipid and blood pressure control therapies in China.	2016	China	Upper middle	East Asia and Pacific
**Wessels**	Is fenofibrate a cost-saving treatment for middle-aged individuals with type 2 diabetes? A South African private-sector perspective.	2010	South Africa	Upper middle	Sub-Saharan Africa
**Singh**	Cost-effectiveness of a fixed dose combination (polypill) in secondary prevention of cardiovascular diseases in India: Within-trial cost-effectiveness analysis of the UMPIRE trial.	2018	India	Lower middle	South Asia
**Sanmukhani**	Statins: cost analysis in Indian scenario from eight major clinical trials.	2010	India	Lower middle	South Asia

Note: This table includes articles that were excluded during the full-text review because cost-effectiveness figures for cholesterol-lowering medications could not be obtained from them, largely due to issues of a lack of disaggregation. However, they do contain cholesterol medication interventions.

Supplementary Table 3: Study characteristics

First Author, publication year	Country, income level, region	Intervention (include if modeled)	Treatment setting (Primary or Secondary)	Outcomes reported	Conclusion (all costs adjusted to 2019 USD)	Currency, year, discount rate, sensitivity analysis performed (y/n)
Amirsadri, 2015	Iran; Upper middle; Middle East and North Africa	A semi-Markov model was used to analyze over the counter 10 mg simvastatin for the primary prevention of myocardial infarction compared to no drug therapy. The base-case uses a 10-year CVD risk of 15% with an annual increase of 0.03% in CVD risk. Second scenario was evaluated in which the probabilities of fatal and non-fatal MI, independent of base-line CVD risk, were sourced from the Isfahan Cohort Study population. In the third scenario, people age 70+ given prescription 10 mg atorvastatin for primary prevention in both the IX and non-IX groups.	P	QALYs and life years gained (LYG)	For the base-case scenario with a 3% discount rate using public sector prices, providing over the counter simvastatin for primary prevention costs US $1,693 per additional QALY gained and US $1,484 per additional life year gained compared to the status quo scenario. Providing over the counter simvastatin was most cost-effective among 45-year-old men with a 10-year CVD risk of 15%.	USD, 2014; Three discount scenarios: 0%, 3 %, and 7.2 % for costs and 3% for effects; Yes
Bautista, 2013	Argentina, Chile, Colombia, Costa Rica, the Dominican Republic, Peru, Puerto Rico, and Venezuela; Multiple; Latin America and the Caribbean	A Markov model was used to analyze the administration of a polypill the following groups: age 55+; people with abdominal obesity per WHO (>88cm in women, >102 in men); people with abdominal obesity per Latin American Consortium of Studies on Obesity (94cm+ women, 91+in men); people with obesity (BMI of 30+); people with metabolic syndrome; and people with an expected 10-year cumulative risk of CVD 15% or more (high risk).	P	QALYs gained	Lifetime CVD risk could be reduced by 15% in women and 21% in men if polypill were used by people with a 10-year CVD risk of 15%+. This would require treating 26% of the population at a cost of US $49 to 52 per QALY. The non-dominated ICERs ranged from US $387 per additional QALY for women at high (15%) CVD risk to 1,100 for men with abdominal obesity (LASO definition), compared to standard care.	USD, 2001 (year not provided, costs from another article that lists year as 2001); 3%; Yes
Borissov, 2017	Bulgaria; Upper middle; Europe and Central Asia	A Markov model analyzed a control scenario that treated familial hypercholesterolemia patients with high-intensity statins (40–80 mg atorvastatin; 20-40 mg rosuvastatin) compared to an IX scenario that treated them with evolucumab, a PCSK9 inhibitor, in addition to the standard of care. Uses novel outcome – effectively treated patient years, which combines length of life with the likelihood of attaining LDL-C lowering goals.	P, S	Effectively treated patient years (ETPYs), life years gained	The total ICERs of adding evolucumab to statin compared to statin alone was US $7,795 per additional ETPY and US $77,952 per life year gained. Addition of evolocumab is associated with a relative reduction in the CVD event rate by 38%.	Bulgarian lev, Not stated, assume year of paper - 2017; 5%; Yes
Briseno, 2010	Mexico; Upper middle; Latin America and the Caribbean	A retrospective, observational study in which patients received drug therapy with either 10 mg/day oral rosuvastatin (RSV) or an oral fixed dose combination of 10 mg ezetimibe and 20 mg simvastatin per day (E/S).	P, S	Achievement of LDL-C goal, percent decrease in LDL-C level	The percentage of patients who reached the LDL-C goals was 46.4% for patients who received RSV, while the proportion for patients who received E/S was 31.4%. The cost of a 1% reduction in LDL-C was $2.02 with RSV and $4.09 with E/S. Treatment with RSV is more effective and less costly.	USD, 2006; No - no future costs/projections made; Yes
Ha, 2011	Vietnam; Lower middle; East Asia and Pacific	This study used the WHO-CHOICE, simulation model, to analyze 12 population- and individual-level IXs for reducing CVD risk factors. Individual-level cholesterol-related IXs	P	DALYs averted	Treating cholesterol concentrations of 5.7 and 6.2 mmol/l+ were the least and second least cost effective IXs in the study, with cost effectiveness	Vietnamese dong, 2007; 3%; Yes

		included treatment with statins for cholesterol concentrations at two different eligibility criteria: 5.7 mmol/l+ and 6.2 mmol/l+. Additionally, combination treatment of beta-blockers, diuretics, statins, and aspirin was modeled for individuals with an absolute risk of CVD with thresholds of 5%, 15%, 25%, and 35% (though this was not in the form of a polypill).			ratios of US $2,306 and 1,713 per DALY, respectively. Combination treatment was more cost-effective, with costs per DALY of US $777, 510, 398, and 325 for the 5%, 15%, 20%, and 35% thresholds, however, these combinations were not in the form of a polypill.	
Khonputsa, 2012	Thailand; Upper middle; East Asia and Pacific	A Markov model was used to analyze single drug interventions and combinations of blood pressure medications, statins, and a polypill. The polypill contained a full-dose statin and three blood pressure-lowering drugs in half-standard doses (diuretic, calcium channel blocker, and ACE inhibitor). The analysis includes cost offsets from averted costs of disease treatments avoided.	P	DALYs averted	The polypill was cost-effective for CVD prevention even in people with 10-year risk of 5–9.9% (CE ratio of US $-367 per DALY averted. Treatment with statins is most cost-effective for those with 10-year CVD risk of 20% with cost per DALY averted of US $842. Among those with a 20% risk, compared to current practice, the ICERs for each additional DALY averted are US $-14,307 and 16,271 for the polypill and statin, respectively.	Thai baht, 2004; 3%; Yes
Konfino, 2017	Argentina; Upper middle; Latin America and Caribbean	A Markov model of adults age 35–84 that simulates three scenarios. Scenario 1 lowers the risk threshold for treatment to >10% according the Framingham Risk Score (FRS); scenario 2 intensifies statin potency under current treatment thresholds; and scenario 3 combines both scenarios by lowering the treatment threshold to ≥10 % FRS and intensifying statin potency.	P, S	QALYs gained	Compared to current treatment guidelines, the ICERs for each additional QALY gained are USD 28,999 for scenario one, 49,293 for scenario two, and 41,979 for scenario three. The figures are all substantially higher than one time Argentina's current GDP per capita.	USD, 2013; 3%; Yes
Kongpak-wattana, 2019	Thailand; Upper middle; East Asia and Pacific	A Markov model used to analyze adding non-statin lipid-modifying agents to statin therapy compared with statins alone. Non-statin lipid-modifying agents considered in this analysis included proprotein convertase subtilisin/kexin type 9 inhibitors (PCSK9i) and ezetimibe.	S	Number of CVD events averted, QALYS	Patients receiving PCSK9i and ezetimibe experienced fewer recurrent CVD events (number needed to treat 17 and 30) and more QALYs (0.168 and 0.096 QALYs gained per person). However, compared to statins alone, the ICER of an additional QALY is US $27,522 for ezetimibe and 547,483 for PCSK9i. Despite the proven effectiveness of PCSK9i and ezetimibe, the costs need to reduce to a much greater extent to be cost-effective in Thailand.	Thai baht, 2018; 3%; Yes
Li, 2018	China; Upper middle; East Asia and Pacific	A simulation model was used to analyze newly diagnosed patients with type 2 diabetes who were assigned to either a control group that did not receive statin therapy, or to an intervention group that received statin therapy.	P	Life years, QALYs	Compared to not providing statins to newly diagnosed diabetic patients, atorvastatin could add 0.08 QALY for an additional US $1,736 (ICER of US $22,866) over a lifetime horizon.	USD, 2017; 5%; Yes
Lin, 2019	China, India, Mexico, Nigeria, South Africa; Multiple; Multiple	A microsimulation Markov model that compared current care in which patients received aspirin, lisinopril, atenolol, and simvastatin in separate individual pills at real-world levels of prescription and use to an intervention scenario in which patients received polypills that contained a fixed-dose combination of the drugs in the current care. Among adults age 30–84 with established atherosclerotic CVD.	S	CVD events averted, DALYs	Adoption of the polypill for secondary prevention compared with current care was projected to have ICERs of US $91 per additional DALY averted in China, 44 in India, 45 in Mexico, 118 in Nigeria, and 30 in South Africa, assuming public sector pharmaceutical prices over a lifetime horizon. The polypill is projected to be cost-effective compared	International dollars, 2017; 3%; Yes

					with current care for secondary prevention in all countries of analysis.	
Megiddo, 2014	India; Lower middle; South Asia	A cohort model that analyzed 5 scenarios for secondary prevention of myocardial infarction: 1) aspirin; 2) aspirin and beta-blockers; 3) aspirin, beta-blockers, and ACEI; 4) aspirin, betablockers, ACEI, and statin; and 5) a hypothetical polypill to be taken once. Polypill treatment is assumed to cost less than the additive cost of all 4 oral medications taken individually, but conservatively, the polypill is not assumed to increase adherence.	S	DALYs averted	The polypill is the dominant strategy with a cost effectiveness ratio of US $2,063. The ICER compared to providing the pills separately is US $-12,644.	USD, 2010; 3%; Yes
Mould-Quevedo, 2014	Brazil and Colombia; Upper middle; Latin America and Caribbean	A Markov cohort model that analyzes primary and secondary prevention. The reference case for primary prevention in Colombia compared atorvastatin 10mg (A10) with rosuvastatin 5mg (R5), and atorvastatin 20mg (A20) with rosuvastatin 10mg (R10). In the Brazilian analysis, A20 was compared with both R10 and rosuvastatin 20mg (R20). For secondary prevention, the comparisons were atorvastatin 40mg (A40) versus R20 and atorvastatin 80mg (A80) versus rosuvastatin 40 mg (R40 for Colombia and A40 versus R40 for Brazil.	P, S	QALYs, life years, CVD events averted	In the Colombian analyses, differences in drug costs between therapies were considerable while outcomes were similar. The ICER per QALY gained for rosuvastatin versus atorvastatin was more than US $770,000 and $220,000 in primary and secondary prevention, respectively. Brazilian analyses found lower ICERs for rosuvastatin at some dose comparisons due to similar pricing between statins. The efficacy advantage of rosuvastatin was minimal, while its acquisition cost was higher, particularly in Colombia.	USD, 2012; 3% for Colombia, 5% Brazil; Yes
Ngalesoni, 2016	Tanzania; Low; Sub-Saharan Africa	A Markov model in which low and moderate risk patients are given BP meds (one or two), then combined with statins. Statins alone are also analyzed. High risk patients receive the same interventions, plus triple BP therapy, then combined with a statin. Very high-risk patients received all the previous interventions, plus adding aspirin, then combined with statins. The CVD risk with existing diabetes model included the same interventions with the addition of biguanide and sulfonylureas.	P	DALYs averted	All statin-only treatment scenarios are dominated and not presented in the study. Among those without diabetes, the ICERs for adding statins to existing BP regimens are US $3,535 per additional DALY averted for low risk, 616 for moderate risk, 676 for high risk, and 726 for very high-risk patients. For those with diabetes, the ICERs for adding statins to existing regimens are US $2,762 for low risk, 1,052 for moderate risk, 1,018 for high risk, and 784 for very high-risk patients per additional DALY averted.	USD, 2012; 3%; Yes
Ortegon, 2012	WHO African region, WHO South-East Asian region; Multiple; Multiple	This analysis uses the WHO-CHOICE model for a total of 123 single and combined intervention strategies (36 for tobacco control, 77 for CVD disease, and 10 for diabetes). The interventions that involve statins only are: individual cholesterol treatment for those with cholesterol levels of 5.7 mmol/l or greater or 6.2 mmol/l or greater, secondary prevention with statins for post-acute ischemic heart disease, and secondary prevention with statins for post-acute stroke. All other interventions involving statins include BP medications not in the form of a polypill.	P	DALYs averted	The cost-effectiveness of individual treatment for cholesterol was lower in South Asia at both the 5.7 and 6.2 thresholds (US $361 and 286, respectively) than in Sub-Saharan Africa (USD 423 and 335, respectively). Treatment of IHD was much less cost effective than treating stroke in Africa (US $1401 compared to 203) than in South Asia (US $226 compared to 108). Risk based treatment strategies were more cost effective but were not in the form of a polypill.	International dollars, 2005; 3%; Yes
Ribeiro, 2015	Brazil; Upper middle; Latin America and the Caribbean	A Markov model is used to evaluate three statin strategies: high-, moderate-, and low-intensity regimens. The three	P, S	QALYs	Low-dose statins were dominated in the primary prevention scenarios and the ICERs were lowest for	International dollars, 2011;

		strategies were analyzed in secondary prevention and in four primary prevention scenarios (5%, 10%, 15%, and 20%, ten-year risk of CVD events).			the intermediate dose statins. In secondary prevention, low dose statins had the lowest ICER.	5%; Yes
Rubinstein, 2010	Argentina; Upper middle; Latin America and Caribbean	A Monte Carlo simulation of treatment of high cholesterol through promotion of low-cholesterol diet and use of statins (atorvastatin 10 mg, 20 mg and 40 mg) and combination therapy (4 pills to approximate a polypill) including hydrochlorothiazide 25 mg, enalapril 10 mg, atorvastatin 10 mg and aspirin 100 mg.	P	DALYs averted	A polypill for people with a 10-year CVD risk of 20% was cost-saving with a cost per DALY of US $1,190 and ICER of US $-151. Cholesterol treatment with statin was found to have a cost per DALY of US $10,043 and ICER of US $8,854.	International dollars, 2007; 3%; Yes
Salomon, 2012	Mexico; Upper middle; Latin America and Caribbean	The analysis uses a population model for 101 treatment strategies, the relevant ones are: statin plus lifestyle modification education for those with total cholesterol >5.7 mmol/l and >6.2mmol/l; treatment with a beta blocker, statin and aspirin, for individuals with 10-year CVD risk of 5%, 15%, 25%, and 35%; however, these were not in the form of a polypill; combination prevention, including hypertension lowering drugs plus lifestyle modification education for those with SBP>140 AND statin plus lifestyle modification for those with cholesterol of > 5.7 mmol/l	P, S	DALYs averted	Individual primary prevention interventions like statin therapy and lifestyle modification resulted in much greater effectiveness, although they were more costly per unit of health benefit than the population-wide strategies included in the analysis. The cost per DALY averted for statin plus lifestyle modification education was US $8,623 for the 5.7 mmol/l threshold and US $6,686 for the 6.2 mmol/l threshold.	International dollars, 2005; 3%; Yes
Sansana-yudh, 2010	Thailand; Upper middle; South Asia	An 8-week RCT was conducted in patients with hypercholesterolemia to compare the efficacy of pitavastatin 1mg per day to atorvastatin 10mg per day.	P, S	Change in lipid levels	Pitavastatin lowered LDL-C levels from baseline by 37% compared with 46% in the atorvastatin group. The monthly cost per percent LDL-C reduction in the pitavastatin group ($0.77) was about 50% lower than the cost in the atorvastatin ($1.56) group. Pitavastatin 1 mg once daily may be an alternative regimen with cost-saving benefits but without a significant decrease in therapeutic benefit or increase in adverse events in patients with hypercholesterolemia.	USD, 2008; No; No
Tolla, 2016	Ethiopia; Low; Sub-Saharan Africa	A multi-state population model (PopMod) analysis of primary prevention of IHD and stroke using 1. simvastatin 40 mg po daily (for total chol >6.2 mmol/l and >5.7 mmol/l) and 2. combination treatment (ASA 100 mg + Hydrochlorothiazide 25 mg + Atenolol 50 mg + Simvastatin 20 mg) for absolute risk of CVD >5%, >15%, >25%, 35%, however, these were not in the form of a polypill.	P, S	DALYs averted	The cost per DALY averted for cholesterol treatment at 5.7 mmol/l is US $620 and the USD 592 for cholesterol treatment at 6.2 mmol/l. For secondary prevention, the cost per DALY for statin therapy for post-acute care for acute ischemic heart disease is US $9,823 and the cost per DALY for post-acute care for stroke is US $2,668.	USD, 2012; 0.03; Yes
Tumanan-Mendoza, 2013	Philippines; Lower middle; East Asia and Pacific	A Markov model comparing four lipid lowering medicines vs placebo. Included medications include simvastatin (40mg/day), pravastatin (40mg/day), atorvastatin (20mg/day), and gemfibrozil (1200mg/day).	S	QALYs	In the analysis using the lower-priced generic counterparts, therapy using 40mg simvastatin daily was the most cost-effective option compared with the other therapies, while pravastatin 40mg daily was the most cost-effective alternative if the<br><br>higher-priced innovator drugs were used. Gemfibrozil was strongly dominated.	Philippine peso, 2010; 3% and 6%; Yes

Wood, 2011	India; Lower middle; South Asia	Cross-sectional and modeling analysis of treatment with blood pressure-lowering medicine, cholesterol-lowering medicines, or a combination pill including a cholesterol-lowering statin, three blood pressure-lowering medications and aspirin.	P, S	CVD events avoided	A polypill would have an incremental cost per CVD event avoided of US $6,582 for individuals with CVD and US $8,598 for those with CVD and at high risk of coronary heart disease, compared to the current treatment levels for blood pressure and cholesterol medications.	USD, 2010; No mention of discounting; No
Yang, 2020	China; Upper middle; East Asia and Pacific	Markov model comparing high-dose rosuvastatin (daily 20mg) vs. ezetimibe (daily 10mg) + moderate-dose rosuvastatin (daily 10mg).	S	QALYs	Adding ezetimibe to the moderate-dose statin in secondary prevention for<br><br>CVD is cost-effective, compared with the high-dose statin, with an ICER per QALY of US $7,271.	Chinese Yuan, 2017; 0% to 5%; Yes

Supplementary Table 4: Average cost effectiveness and incremental cost effectiveness measures per DALY averted, 2019 USD

First Author	Country	WB Income Level	Region	IX Description	ACER or ICER	Intervention Subgroup	Cost per DALY Averted in 2019 USD
Ha	Vietnam	Lower middle	East Asia and Pacific	In addition to population-level interventions not described here, the study included individual treatment with statins for cholesterol concentrations at two different eligibility criteria: 5.7 mmol/l or greater and 6.2 mmol/l or greater. Additionally, combination treatment of BP drugs, statins, and aspirin was modeled for individuals with different risk levels though these were not in a polypill form.	ACER	Treating those with cholesterol of 5.7 mmol/l or greater with simvastatin 40 mg per day	2,405.78
						Treating those with cholesterol of 6.2 mmol/l or greater with simvastatin 40 mg per day	1,712.77
					ICER	Treating those with cholesterol of 6.2 mmol/l or greater with simvastatin 40 mg per day, compared to treating those with cholesterol of 5.7 mmol/l or greater.	3,812.43
Khonputsa	Thailand	Upper middle	East Asia and Pacific	Blood pressure medications, statins, and a polypill were included in the analysis for primary prevention of CVD. The analysis was conducted for single drug interventions and combinations of drugs from different classes. The polypill contained a full-dose statin and three blood pressure-lowering drugs in half-standard doses (diuretic, calcium channel blocker, and ACE inhibitor).	ACER	Current practice	10,099.33
						Treatment with polypill for those with 10-year CVD risk of 5-9.9%	-367.25
						Treatment with statin for those with 10-year CVD risk of 5-9.9%	3,107.49
						Treatment with polypill for those with 10-year CVD risk of 10-19.9%	-591.90
						Treatment with statin for those with 10-year CVD risk of 10-19.9%	1,923.68
						Treatment with polypill for those with 10-year CVD risk of 20+%	-748.10
						Treatment with statin for those with 10-year CVD risk of 20+%	841.61
					ICER	Treatment with polypill for those with 10-year CVD risk of 5-9.9%, compared to current practice	-6,348.15
						Treatment with statin for those with 10-year CVD risk of 5-9.9%, compared to current practice	23,084.19
						Treatment with polypill for those with 10-year CVD risk of 10-19.9%, compared to current practice	-8,977.18
						Treatment with statin for those with 10-year CVD risk of 10-19.9%, compared to current practice	19,135.57
						Treatment with polypill for those with 10-year CVD risk of 20+%, compared to current practice	-14,307.39
						Treatment with statin for those with 10-year CVD risk of 20+%, compared to current practice	16,271.14
Lin	China, India, Mexico, Nigeria, South Africa	Multiple	Multiple	Current care – patients received aspirin 75 mg, lisinopril 10 mg, atenolol 50 mg, and simvastatin 40 mg in separate individual pills at real-world levels of prescription and use for secondary prevention of CVD. Intervention - polypills that contained a fixed-dose combination of the drugs in the current care scenario were given to patients.	ACER		
						Polypill, China, public sector pharmaceutical prices	130.31
						Polypill, India, public sector pharmaceutical prices	18.20
						Polypill, Mexico, public sector pharmaceutical prices	87.15
						Polypill, Nigeria, public sector pharmaceutical prices	143.03
						Polypill, South Africa, public sector pharmaceutical prices	102.71
						Polypill, China, retail market pharmaceutical prices	175.56
						Polypill, India, retail market pharmaceutical prices	24.42
						Polypill, Mexico, retail market pharmaceutical prices	120.54
						Polypill, Nigeria, retail market pharmaceutical prices	164.41
						Polypill, South Africa, retail market pharmaceutical prices	133.52
					ICER	Polypill, China, public sector pharmaceutical prices, compared to current care	91.49
						Polypill, India, public sector pharmaceutical prices, compared to current care	43.63
						Polypill, Mexico, public sector pharmaceutical prices, compared to current care	44.96
						Polypill, Nigeria, public sector pharmaceutical prices, compared to current care	117.93
						Polypill, South Africa, public sector pharmaceutical prices, compared to current care	30.24
						Polypill, China, retail market pharmaceutical prices, compared to current care	438.62
						Polypill, India, retail market pharmaceutical prices, compared to current care	100.55
						Polypill, Mexico, retail market pharmaceutical prices, compared to current care	307.05
						Polypill, Nigeria, retail market pharmaceutical prices, compared to current care	276.29
						Polypill, South Africa, retail market pharmaceutical prices, compared to current care	235.13
Megiddo	India	Lower	South	The analysis includes 5 scenarios for secondary	ACER		

		middle	Asia	prevention of myocardial infarction: 1) aspirin (75 mg once daily); 2) aspirin and beta-blockers (75 mg once daily and 50 mg twice daily, respectively); 3) aspirin, beta-blockers, and ACEI (75 mg once daily, 50 mg twice daily, and 5 mg once daily, respectively); 4) aspirin, betablockers, ACEI, and statin (75 mg once daily, 50 mg twice daily, 5 mg once daily, and 10 mg once daily, respectively); and 5) a hypothetical polypill to be taken once. Polypill treatment is assumed to cost less than the additive cost of all 4 oral medications taken individually, but conservatively, the polypill is not assumed to increase adherence.		Polypill	2,063.49
					ICER	Polypill, compared to aspirin + BB + ACE inhibitor + statin	-12,633.58
					ICER	Low CVD risk, no existing diabetes, ACE inhibitor + diuretic + statin, compared to without statin	3,535.42
						Moderate CVD risk, no existing diabetes, ACE inhibitor + diuretic + statin, compared to without statin	60.13
						High CVD risk, no existing diabetes, ACE inhibitor + calcium channel blocker + diuretic + statin, compared to without statin	675.91
						Very high CVD risk, no existing diabetes, ACE inhibitor + calcium channel blocker + diuretic + aspirin + statin, compared to without statin	726.01
						Low CVD risk, existing diabetes, biguanide + sulfonylureas + ACE inhibitor + calcium channel blocker + statin, compared to without statin	2,761.52
						Moderate CVD risk, existing diabetes, biguanide + sulfonylureas + +ACE inhibitor + calcium channel blocker + statin, compared to without statin	1,052.27
						High CVD risk, existing diabetes, biguanide + sulfonylureas + ACE inhibitor + calcium channel blocker + statin, compared to without statin	1,017.76
						Very high CVD risk, existing diabetes, biguanide + sulfonylureas + ACE inhibitor + CCB + aspirin + statin, compared to without statin	783.92
Ortegon	WHO African region, WHO South East Asian region	Multiple	Multiple	A total of 123 single and combined interventions strategies were assessed, (36 for tobacco control, 77 for CVD disease, and 10 for diabetes). The interventions that involve statins only are: individual cholesterol treatment for those with a cholesterol level of 5.7 mmol/l or greater, treatment for those with levels of 6.2 mmol/l or greater, secondary prevention with statins for post-acute ischemic heart disease, and secondary prevention with statins for post-acute stroke. All other interventions involving statins include BP medications not in the form of a polypill.	ACER	Individual cholesterol treatment (>5.7 mmol/l) Africa	422.77
						Individual cholesterol treatment (>5.7 mmol/l) South East Asia	361.09
						Individual cholesterol treatment (>6.2 mmol/l) Africa	335.22
						Individual cholesterol treatment (>6.2 mmol/l) South East Asia	286.11
						Treatment of post-acute IHD with statins, Africa	1,401.36
						Treatment of post-acute IHD with statins, South East Asia	225.78
						Treatment of post-acute stroke with statins, Africa	203.32
						Treatment of post-acute stroke with statins, South East Asia	108.07
Rubinstein	Argentina	Upper middle	Latin America and Caribbean	Treatment of high cholesterol through promotion of low-cholesterol diet and use of statins (atorvastatin 10 mg, 20 mg and 40 mg) among adults 35 and older with high cholesterol, and combination therapy (4 pills to approximate a polypill) including hydrochlorothiazide 25 mg, enalapril 10 mg, atorvastatin 10 mg and aspirin 100 mg among adults 35 and older with risk of >20% 10-year CVD risk.	ACER	Pharmacological therapy of high cholesterol	10,042.72
						Treatment targeted to persons with an absolute risk above 20% in 10 years (modified polypill strategy)	1,190.20
					ICER	Pharmacological therapy of high cholesterol	8,853.83
						Treatment targeted to persons with an absolute risk above 20% in 10 years (modified polypill strategy)	-151.20
						Statin plus lifestyle modification, delivered by physicians to individuals with serum cholesterol concentration > 220 mg/dl (> 5.7 mmol/l), 3% discounting and age weighting	19,855.58
						Statin plus lifestyle modification, delivered by physicians to individuals with serum cholesterol concentration > 240 mg/dl (> 6.2 mmol/l), 3% discounting and age weighting	8,232.70
						Statin plus lifestyle modification, delivered by physicians to individuals with serum cholesterol concentration > 220 mg/dl (> 5.7 mmol/l), 3% discounting and no age weighting	8,623.49
						Statin plus lifestyle modification, delivered by physicians to individuals with serum cholesterol concentration > 240 mg/dl (> 6.2 mmol/l), 3% discounting and no age weighting	6,685.67
Tolla	Ethiopia	Low	Sub-Saharan Africa	Secondary prevention using a statin for ischemic heart disease and stroke. Primary prevention of IHD and stroke using 1. Simvastatin 40 mg po daily (for total chol >6.2 mmol/l and >5.7 mmol/l) and 2. combination treatment (ASA 100 mg + Hydrochlorothiazide 25 mg + Atenolol 50 mg + Simvastatin 20 mg) for absolute risk of CVD >5%, >15%, >25%, 35%	ACER	Cholesterol lowering treatment for total chol. >6.2 mmol/l	592.39
						Cholesterol lowering treatment for total chol. >5.7 mmol/l	620.23
						Statin, secon. prev. stroke	2668
						Statin, secon. prev. IHD	9823
					ICER	Cholesterol lowering treatment for total chol >5.7 mmol/l, compared to >6.2 mmol/l	642.93

Supplementary Table 5: Average cost effectiveness and incremental cost effectiveness measures per QALY gained, 2019 USD

First Author	Country	WB Income Level/Region	Treatment (IX) Description	ACER or ICER	IX Subgroup	Cost per QALY Gained in 2019 USD
Amirsadri	Iran	Upper middle/ Middle East and North Africa	Use of over the counter (OTC) simvastatin 10mg (a low dose statin) for the primary prevention of myocardial infarction (MI) compared to no drug-therapy. The base-case takes into account a 10-year CVD risk of 15% with an annual increase of 0.03% in CVD risk. A second scenario was evaluated in which the probabilities of fatal and non-fatal MI, independent of base-line CVD risk, were sourced from the Isfahan Cohort Study (ICS) population. In a third scenario, people aged 70 or more were given prescription statin (atorvastatin 10 mg) for primary prevention in both the IX and nonIX groups. Three different discounting scenarios were also considered.	ACER	Simvastatin, discount rate 0%, public prices, IX scenario	26.30
					Simvastatin, discount rate 0%, private prices, IX scenario	33.48
					Simvastatin with ICS mort/morb trans. prob. data, discount rate 0%, public prices, IX scenario	27.75
					Simvastatin with ICS mort/morb trans. prob. data, discount rate 0%, private prices, IX scenario	36.79
					Simvastatin with both control and IX group taking 10 mg atorvastatin at age 70+, discount rate 0%, public prices, IX scenario	30.96
					Simvastatin with both control and IX group taking 10 mg atorvastatin at age 70+, discount rate 0%, private prices, IX scenario	44.62
					Simvastatin, discount rate 3%, public prices, IX scenario	24.83
					Simvastatin, discount rate 3%, private prices, IX scenario	30.83
					Simvastatin with ICS mort/morb trans. prob. data, discount rate 3%, public prices, IX scenario	25.72
					Simvastatin with ICS mort/morb trans. prob. data, discount rate 3%, private prices, IX scenario	32.87
					Simvastatin with both control and IX group taking 10 mg atorvastatin at age 70+, discount rate 3%, public prices, IX scenario	27.62
					Simvastatin with both control and IX group taking 10 mg atorvastatin at age 70+, discount rate 3%, private prices, IX scenario	37.51
					Simvastatin, discount rate 7.2% for costs and 3% for effects, public prices, IX scenario	14.66
					Simvastatin, discount rate 7.2% for costs and 3% for effects, private prices, IX scenario	17.70
					Simvastatin with ICS mort/morb trans. prob. data, discount rate 7.2% for costs and 3% for effects, public prices, IX scenario	14.97
					Simvastatin with ICS mort/morb trans. prob. data, discount rate 7.2% for costs and 3% for effects, private prices, IX scenario	18.32
					Simvastatin with both control and IX group taking 10 mg atorvastatin at age 70+, discount rate 7.2% for costs and 3% for effects, public prices, IX scenario	15.43
					Simvastatin with both control and IX group taking 10 mg atorvastatin at age 70+, discount rate 7.2% for	19.56

					costs and 3% for effects, private prices, IX scenario	
				ICER	Simvastatin, discount rate 0%, public prices, compared to no-drug scenario	1,202.39
					Simvastatin, discount rate 0%, private prices, compared to no-drug scenario	1,057.95
					Simvastatin with ICS mort/morb trans. prob. data, discount rate 0%, public prices, compared to no-drug scenario	955.72
					Simvastatin with ICS mort/morb trans. prob. data, discount rate 0%, private prices, compared to no-drug scenario	812.81
					Simvastatin with both control and IX group taking 10 mg atorvastatin at age 70+, discount rate 0%, public prices, compared to no-drug scenario	1,081.47
					Simvastatin with both control and IX group taking 10 mg atorvastatin at age 70+, discount rate 0%, private prices, compared to no-drug scenario	965.50
					Simvastatin, discount rate 3%, public prices, compared to no-drug scenario	1,693.04
					Simvastatin, discount rate 3%, private prices, compared to no-drug scenario	1,519.45
					Simvastatin with ICS mort/morb trans. prob. data, discount rate 3%, public prices, compared to no-drug scenario	1,413.84
					Simvastatin with ICS mort/morb trans. prob. data, discount rate 3%, private prices, compared to no-drug scenario	1,244.15
					Simvastatin with both control and IX group taking 10 mg atorvastatin at age 70+, discount rate 3%, public prices, compared to no-drug scenario	1,648.77
					Simvastatin with both control and IX group taking 10 mg atorvastatin at age 70+, discount rate 3%, private prices, compared to no-drug scenario	1,494.79
					Simvastatin, discount rate 7.2% for costs and 3% for effects, public prices, compared to no-drug scenario	1,063.86
					Simvastatin, discount rate 7.2% for costs and 3% for effects, private prices, compared to no-drug scenario	973.68
					Simvastatin with ICS mort/morb trans. prob. data, discount rate 7.2% for costs and 3% for effects, public prices, compared to no-drug scenario	905.58
					Simvastatin with ICS mort/morb trans. prob. data, discount rate 7.2% for costs and 3% for effects, private prices, compared to no-drug scenario	823.05
					Simvastatin with both control and IX group taking 10 mg atorvastatin at age 70+, discount rate 7.2% for costs and 3% for effects, public prices, compared to no-drug scenario	1,163.31
					Simvastatin with both control and IX group taking 10 mg atorvastatin at age 70+, discount rate 7.2% for costs and 3% for effects, private prices, compared to no-drug scenario	1,070.06
Bautista	Argentina, Chile, Colombia,	Multiple/ Latin America and	Administration of the polypill for primary prevention to people in the following target groups: people age 55 or older; people with	ACER	No IX (no polypill), men	29.59
					No IX (no polypill), women	36.03

	Costa Rica, the Dominican Republic, Peru, Puerto Rico, and Venezuela	Caribbean	abdominal obesity as defined by the WHO (waist circumference >88 cm in women, >102 cm in men); people with abdominal obesity as defined by the Latin American Consortium of Studies on Obesity (waist circumference of 94 cm or more in women, 91 or more cm in men); people with obesity (BMI of 30 or higher); people with metabolic syndrome as defined by the Adult Treatment Panel III report; and people with an expected 10-year risk of CVD 15% or more (high risk).		Polypill for men aged 55 years and older	40.40
					Polypill for women at high risk - 10 year CVD risk of 15% or greater	45.20
					Polypill for men at high risk - 10-year CVD risk of 15% or greater	48.39
					Polypill for men with abdominal obesity (LASO definition - 91 cm or greater)	55.54
					Polypill for women with abdominal obesity (WHO definition - 88 cm or greater)	70.40
				ICER	Polypill for women at high risk - 10-year CVD risk of 15% or greater, compared to no polypill (standard care)	386.51
					Polypill for men aged 55 years and older, compared to no polypill (standard care)	646.99
					Polypill for men at high risk - 10-year CVD risk of 15% or greater, compared to no polypill (standard care)	853.02
					Polypill for women with abdominal obesity (WHO definition - 88 cm or greater), compared to no polypill (standard care)	1,096.22
					Polypill for men with abdominal obesity (LASO definition - 91 cm or greater), compared to no polypill (standard care)	1,100.12
Konfino	Argentina	Upper middle/ Latin America and Caribbean	Current guidelines - those w/o a history of CVD, treated 50% of those with 20%+ CVD risk or diabetes. For those with a history of CVD, assumed 15% already on a statin and treated 50% of remaining. Newly treated assumed to be on a moderate-dose statin, with decrease in mean LDL-C of 40%. Variation 1 – expansion of primary prevention. Treated the population described for the current guidelines scenario and expanded the primary prevention population to include 50% of treatment-naïve individuals with a risk of 10–20 %, assuming treatment with a moderate-dose statin. Variation 2 – use of high-potency statins with current guideline's target pop., with a decrease in LDL-C of 55%. Variation 3 – expansion of primary prevention and use of high-potency statin with high-risk population (comb. of IXs in scenarios 1 and 2), with those qualifying under current national guidelines treated with a high-potency statin and those with no history of CVD but an FRS score of 10–20 % treated with a moderate-dose statin.	ACER	Current guidelines	7.57
					Variation 1: Lower primary prevention treatment threshold	12.21
					Variation 2: Use high-potency statin use for high-risk patients	18.00
					Variation 3: Use high-potency statins for those at risk under current guidelines, use moderate-potency with those 10-20% risk	22.60
				ICER	Variation 1: Lower primary prevention treatment threshold, compared to current guidelines	28,999.27
					Variation 3: Use high-potency statins for those at risk under current guidelines, use moderate-potency with those 10-20% risk, compared to current guidelines	41,979.21
					Variation 2: Use high-potency statin use for high-risk patients, compared to current guidelines	49,392.25
Kongpak-	Thailand	Upper	Non-statin lipid-modifying agents added to	ACER	Statin alone (healthcare system perspective)	1,286.21

wattana		middle/ East Asia and Pacific	statin therapy compared with statins alone for patients aged 62 and older with existing CVD. Non-statin lipid-modifying agents considered in this analysis included proprotein convertase subtilisin/kexin type 9 inhibitors (PCSK9i) and ezetimibe.		Statin alone (societal perspective)	1,494.70
					Ezetimibe added to statin (healthcare system perspective)	1,512.77
					Ezetimibe added to statin (societal perscpectiv)	1,720.90
					PCSK9 inhibitor added to statin (healthcare system perspective)	9,537.07
					PCSK9 inhibitor added to statin (societal perspective)	9,744.93
				ICER	Ezetimibe added to statin (healthcare system perspective)	27,354.32
					Ezetimibe added to statin (societal perscpectiv)	27,521.57
					PCSK9 inhibitor added to statin (healthcare system perspective)	547,316.14
					PCSK9 inhibitor added to statin (societal perspective)	547,483.23
Li	China	Upper middle/ East Asia and Pacific	Primary prevention with statin treatment amount newly diagnosed patients with type 2 diabetes, assigned to either a control group that did not receive statin therapy, or to a IX group that received statin therapy.	CE	Control group (no statin therapy given to diabetic patients)	1,110.20
					IX group (statin therapy provided to diabetic patients)	1,269.64
				ICER	IX group (statin therapy provided to diabetic patients), compared to control group	21,837.54
Mould-Quevedo	Brazil and Colombia	Upper middle/ Latin America and Caribbean	For the reference case analysis for primary prevention in Colombia, atorvastatin 10mg (A10) was compared with rosuvastatin 5mg (R5), and atorvastatin 20mg (A20) was compared with rosuvastatin 10mg (R10). In the Brazilian analysis, A20 was compared with both R10 and rosuvastatin 20mg (R20). For secondary prevention, the comparisons were atorvastatin 40mg (A40) versus R20 and atorvastatin 80mg (A80) versus rosuvastatin 40 mg (R40 for Colombia and A40 versus R40 for Brazil.	ACER	Brazil, primary prevention, atorvastatin 20 mg	251.64
					Brazil, primary prevention, rosuvastatin 10 mg	253.47
					Brazil, primary prevention, rosuvastatin 20 mg	93.09
					Brazil, secondary prevention, atorvastatin 40 mg	509.99
					Brazil, secondary prevention, rosuvastatin 40 mg	575.64
					Colombia, primary prevention, atorvastatin 10 mg	368.52
					Colombia, primary prevention, atorvastatin 20 mg	517.26
					Colombia, primary prevention, rosuvastatin 10 mg	825.85
					Colombia, primary prevention, rosuvastatin 5 mg	725.94
					Colombia, secondary prevention, atorvastatin 40 mg	1,371.96
					Colombia, secondary prevention, atorvastatin 80 mg	1,512.32
					Colombia, secondary prevention, rosuvastatin 20 mg	1,755.47
					Colombia, secondary prevention, rosuvastatin 40 mg	2,000.35

				ICER	Brazil, primary prevention, rosuvastatin 10 mg, compared to atorvastatin 20 mg	4,827.10
					Brazil, secondary prevention, rosuvastatin 40 mg, compared to atorvastatin 40 mg	18,674.81
					Brazil, primary prevention, rosuvastatin 20 mg, compared to atorvastatin 20 mg	150,458.52
					Colombia, secondary prevention, rosuvastatin 20 mg, compared to atorvastatin 40 mg	224,559.79
					Colombia, secondary prevention, rosuvastatin 40 mg, compared to atorvastatin 80 mg	287,474.66
					Colombia, primary prevention, rosuvastatin 10 mg, compared to atorvastatin 20 mg	779,687.37
					Colombia, primary prevention, rosuvastatin 5 mg, compared to atorvastatin 10 mg	903,481.60
Ribeiro	Brazil	Upper middle/<br><br>Latin America and Caribbean	Three statin strategies were evaluated: high-, moderate-, and low-intensity regimens. The three strategies were analyzed in secondary prevention (patients 45 to 85 who had suffered stable angina, myocardial infarction or stroke) and in four primary prevention scenarios (5%, 10%, 15%, and 20%, ten-year risk of CVD events).	ACER	Primary prevention, 5% risk, no statin treatment	117.31
					Primary prevention, 5% risk, low dose statin treatment	147.47
					Primary prevention, 5% risk, moderate dose statin treatment	167.13
					Primary prevention, 10% risk, no statin treatment	258.78
					Primary prevention, 10% risk, low dose statin treatment	279.24
					Primary prevention, 10% risk, moderate dose statin treatment	291.63
					Primary prevention, 5% risk, high dose statin treatment	431.76
					Primary prevention, 10% risk, high dose statin treatment	547.69
					Primary prevention, 15% risk, no statin treatment	922.59
					Primary prevention, 15% risk, low dose statin treatment	940.04
					Primary prevention, 15% risk, moderate dose statin treatment	950.95
					Primary prevention, 20% risk, no statin treatment	1,117.38
					Primary prevention, 20% risk, low dose statin treatment	1,130.19
					Primary prevention, 20% risk, moderate dose statin treatment	1,138.29
					Primary prevention, 15% risk, high dose statin treatment	1,206.23
					Primary prevention, 20% risk, high dose statin treatment	1,389.89
					Secondary prevention, no statin treatment	2,557.19
					Secondary prevention, low dose statin treatment	2,582.28
					Secondary prevention, moderate dose statin treatment	2,606.86

					Secondary prevention, high dose statin treatment	2,913.53
				ICER	Primary prevention, 20% risk, moderate dose statin treatment, compared to no statin treatment	2,530.98
					Primary prevention, 20% risk, low dose statin treatment, compared to no statin treatment	2,958.29
					Primary prevention, 15% risk, moderate dose statin treatment, compared to no statin treatment	3,358.89
					Secondary prevention, low dose statin treatment, compared to no statin treatment	3,551.12
					Secondary prevention, moderate dose statin treatment, compared to no statin treatment	3,757.73
					Primary prevention, 15% risk, low dose statin treatment, compared to no statin treatment	3,903.29
					Primary prevention, 10% risk, moderate dose statin treatment, compared to no statin treatment	4,545.28
					Primary prevention, 10% risk, low dose statin treatment, compared to no statin treatment	5,577.60
					Secondary prevention, high dose statin treatment, compared to no statin treatment	10,138.29
					Primary prevention, 5% risk, moderate dose statin treatment, compared to no statin treatment	10,699.13
					Primary prevention, 20% risk, high dose statin treatment, compared to no statin treatment	13,226.54
					Primary prevention, 5% risk, low dose statin treatment, compared to no statin treatment	16,085.68
					Primary prevention, 15% risk, high dose statin treatment, compared to no statin treatment	17,263.51
					Primary prevention, 10% risk, high dose statin treatment, compared to no statin treatment	23,571.47
					Primary prevention, 5% risk, high dose statin treatment, compared to no statin treatment	47,913.66
Tumanan-Mendoza	Philippines	Lower middle/ East Asia and Pacific	Comparison of four lipid lowering medicines vs. placebo. Included medications include simvastatin (40mg/day), pravastatin (40mg/day), atorvastatin (20mg/day), and gemfibrozil (1200mg/day) for secondary prevention.	ACER	Simvastatin 40mg/d, 6% discount rate, base case (lowest cost, starting age 35 years)	376.11
					Atorvastatin 20mg/d, 6% discount rate, base case (lowest cost, starting age 35 years)	471.70
					Pravastatin 40mg/d, 6% discount rate, base case (lowest cost, starting age 35 years)	490.20
					Simvastatin 40mg/d, 6% discount rate, sensitivity analysis (highest cost of drugs)	490.20
					Pravastatin 40mg/d, 6% discount rate, sensitivity analysis (highest cost of drugs)	569.16
					Simvastatin 40mg/d, 3% discount rate, base case (lowest cost, starting age 35 years)	585.76
					Simvastatin 40mg/d, 6% discount rate, sensitivity analysis (starting age=45 years)	585.76
					Gemfibrozil 1200mg/d, 6% discount rate, base case (lowest cost, starting age 35 years)	613.58
					Atorvastatin 20mg/d, 6% discount rate, sensitivity analysis (highest cost of drugs)	632.09
					Gemfibrozil 1200mg/d, 3% discount rate, base case (lowest cost, starting age 35 years)	681.38

					Gemfibrozil 1200mg/d, 6% discount rate, sensitivity analysis (starting age=45 years)	681.38
					Atorvastatin 20mg/d, 3% discount rate, base case (lowest cost, starting age 35 years)	699.88
					Atorvastatin 20mg/d, 6% discount rate, sensitivity analysis (starting age=45 years)	699.88
					Simvastatin 40mg/d, 6% discount rate, sensitivity analysis (highest cost in all centers)	740.45
					Simvastatin 40mg/d, 3% discount rate, sensitivity analysis (starting age=45 years, highest cost in all)	740.45
					Pravastatin 40mg/d, 6% discount rate, sensitivity analysis (highest cost in all centers)	819.38
					Pravastatin 40mg/d, 3% discount rate, sensitivity analysis (starting age=45 years, highest cost in all)	819.38
					Pravastatin 40mg/d, 3% discount rate, base case (lowest cost, starting age 35 years)	823.24
					Pravastatin 40mg/d, 6% discount rate, sensitivity analysis (starting age=45 years)	823.24
					Atorvastatin 20mg/d, 6% discount rate, sensitivity analysis (highest cost in all centers)	882.31
					Atorvastatin 20mg/d, 3% discount rate, sensitivity analysis (starting age=45 years, highest cost in all)	882.31
					Gemfibrozil 1200mg/d, 6% discount rate, sensitivity analysis (highest cost of drugs)	2,346.82
					Gemfibrozil 1200mg/d, 6% discount rate, sensitivity analysis (highest cost in all centers)	2,597.07
					Gemfibrozil 1200mg/d, 3% discount rate, sensitivity analysis (starting age=45 years, highest cost in all)	2,597.07
				ICER	Atorvastatin 20mg/d, 6% discount rate, sensitivity analysis (highest cost of drugs), comparison to Pravastatin 40 mg/d	1,487.10
					Atorvastatin 20mg/d, 3% discount rate, sensitivity analysis (starting age=45 years, highest cost in all), comparison to Pravastatin 40mg/d	1,681.50
					Atorvastatin 20mg/d, 6% discount rate, sensitivity analysis (highest cost in all centers), comparison to Pravastatin 40 mg/d	1,737.32
					Atorvastatin 20mg/d, 6% discount rate, base case (lowest cost, starting age 35 years), comparison to Simvastatin 40mg/d	3,166.00
					Atorvastatin 20mg/d, 3% discount rate, base case (lowest cost, starting age 35 years), comparison to Simvastatin 40mg/d	3,200.76
					Atorvastatin 20mg/d, 6% discount rate, sensitivity analysis (starting age=45 years), comparison to Simvastatin 40mg/d	3,393.32
Yang	China	Upper middle/ East Asia and Pacific	Comparison of high-dose rosuvastatin (daily 20mg) vs. ezetimibe (daily 10mg) + moderate-dose rosuvastatin (daily 10mg) for secondary prevention of CVD.	ACER	High-dose rosuvastatin (20 mg) regimen	1,590.81
					Combination regimen of moderate-dose rosuvastatin (10mg) with ezetimibe (10mg)	1,875.04
				ICER	Combination regimen of moderate-dose rosuvastatin (10mg) with ezetimibe (10mg) compared to high-dose rosuvastatin (20 mg)	7,271.41

Supplementary Table 6: Average cost effectiveness and incremental cost effectiveness measures per CVD events averted, 2019 USD

First Author	Country	WB Income Level	Region	IX Description	IC or ICER	Intervention Subgroup	Cost in 2019 USD
Borissov	Bulgaria	Upper middle	Europe and Central Asia	The standard of care scenario (control) was treating heterozygous familial hypercholesterolemia (HeFH) patients (with or without a history of CVD) with high-intensity statins (40–80 mg atorvastatin; 20–40 mg rosuvastatin). The intervention scenario involves treating HEFH patients with evolucumab, a PCSK9 inhibitor, in addition to the standard of care.	CE	Status quo treatment (high-intensity statins) for patients with HeFH	4,005.23
						Evolocumab plus status quo treatment (high-intensity statins) for patients with HeFH	46,514.17
					ICER	Evolocumab plus status quo treatment (high-intensity statins) for patients with HeFH, compared to status quo treatment alone	-65,311.14
Kongpak-wattana	Thailand	Upper middle	East Asia and Pacific	Non-statin lipid-modifying agents added to statin therapy compared with statins alone. Non-statin lipid-modifying agents considered in this analysis included proprotein convertase subtilisin/kexin type 9 inhibitors (PCSK9i) and ezetimibe.	CE	Ezetimibe added to statin (healthcare system perspective)	506,363.81
						Ezetimibe added to statin (societal perscpectiv)	576,032.36
						PCSK9 inhibitor added to statin (healthcare system perspective)	1,828,156.95
						PCSK9 inhibitor added to statin (societal perspective)	1,868,003.11
						Polypill, China, public sector pharmaceutical prices	4,647.37
						Polypill, India, public sector pharmaceutical prices	604.64
						Polypill, Mexico, public sector pharmaceutical prices	3,580.39
						Polypill, Nigeria, public sector pharmaceutical prices	6,356.98
						Polypill, South Africa, public sector pharmaceutical prices	4,610.37
						Polypill, China, retail market pharmaceutical prices	6,261.26
						Polypill, India, retail market pharmaceutical prices	811.35
						Polypill, Mexico, retail market pharmaceutical prices	4,952.51
						Polypill, Nigeria, retail market pharmaceutical prices	7,307.04
						Polypill, South Africa, retail market pharmaceutical prices	5,993.48
					ICER	Polypill, China, public sector pharmaceutical prices, compared to current care	1,585.29
						Polypill, India, public sector pharmaceutical prices, compared to current care	614.11
						Polypill, Mexico, public sector pharmaceutical prices, compared to current care	846.30
						Polypill, Nigeria, public sector pharmaceutical prices, compared to current care	2,424.04
						Polypill, South Africa, public sector pharmaceutical prices, compared to current care	640.39
						Polypill, China, retail market pharmaceutical prices, compared to current care	7,599.88
						Polypill, India, retail market pharmaceutical prices, compared to current care	1,415.12
						Polypill, Mexico, retail market pharmaceutical prices, compared to current care	5,780.29
						Polypill, Nigeria, retail market pharmaceutical prices, compared to current care	5,679.37
						Polypill, South Africa, retail market pharmaceutical prices, compared to current care	5,276.06
Mould-Quevedo	Brazil and Colombia	Upper middle	Latin America and Caribbean	For the reference case analysis for primary prevention in Colombia, atorvastatin 10mg (A10) was compared with rosuvastatin 5mg (R5), and atorvastatin 20mg (A20) was compared with rosuvastatin 10mg (R10). In the Brazilian analysis, A20 was compared with both R10 and rosuvastatin 20mg (R20). For secondary prevention, the comparisons were atorvastatin 40mg	CE	Colombia, primary prevention, atorvastatin 10 mg	16,472.64
						Colombia, primary prevention, rosuvastatin 5 mg	32,473.89
						Colombia, primary prevention, atorvastatin 20 mg	23,138.90
						Colombia, primary prevention, rosuvastatin 10 mg	36,942.81
						Colombia, secondary prevention, atorvastatin 40 mg	14,800.04
						Colombia, secondary prevention, rosuvastatin 20 mg	19,199.02
						Colombia, secondary prevention, atorvastatin 80 mg	16,559.00
						Colombia, secondary prevention, rosuvastatin 40 mg	21,953.21
						Brazil, primary prevention, atorvastatin 20 mg	12,078.74
						Brazil, primary prevention, rosuvastatin 10 mg	12,166.65

				(A40) versus R20 and atorvastatin 80mg (A80) versus rosuvastatin 40 mg (R40 for Colombia and A40 versus R40 for Brazil.		Brazil, primary prevention, rosuvastatin 20 mg	18,888.78
						Brazil, secondary prevention, atorvastatin 40 mg	5,580.44
						Brazil, secondary prevention, rosuvastatin 40 mg	6,456.22
					ICER	Colombia, primary prevention, rosuvastatin 5 mg, compared to atorvastatin 10 mg	470,681.74
						Colombia, primary prevention, rosuvastatin 10 mg, compared to atorvastatin 20 mg	4,252,831.71
						Colombia, secondary prevention, rosuvastatin 20 mg, compared to atorvastatin 40 mg	837,786.28
						Colombia, secondary prevention, rosuvastatin 40 mg, compared to atorvastatin 80 mg	1,073,001.32
						Brazil, primary prevention, rosuvastatin 10 mg, compared to atorvastatin 20 mg	19,288.35
						Brazil, primary prevention, rosuvastatin 20 mg, compared to atorvastatin 20 mg	601,562.39
						Brazil, secondary prevention, rosuvastatin 40 mg, compared to atorvastatin 40 mg	40,958.52
Wood	India	Lower middle	South Asia	Treatment of all patients with CVD or at high risk of CHD with blood pressure-lowering medicine, cholesterol-lowering medicines, or a combination pill including a cholesterol-lowering statin, three blood pressure-lowering medications and aspirin. An alternative scenario analyses the cost-effectiveness of single-risk factor-based treatment of treating all patients with high blood pressure or high cholesterol.	CE	Status quo treatment at baseline treatment patterns for blood-pressure and cholesterol lowering medications	4,591.26
						Combination pill for individuals with established CVD	5,456.52
						Combination pill for individuals with established CVD and at high risk of CHD	6,245.57
						Cholesterol lowering medications for individuals with dyslipidemia (single risk factor based treatment)	29,697.84
					ICER	Combination pill for individuals with established CVD, compared to status quo	6,581.81
						Combination pill for individuals with established CVD and at high risk of CHD, compared to status quo	8,597.58

Supplementary Table 7: Cost effectiveness and incremental cost effectiveness measures per life year gained, 2019 USD

First Author	Country	WB Income Level	Region	IX Description	ACER or ICER	Intervention Subgroup	Cost in 2019 USD
Amirsadri	Iran	Upper middle	Middle East and North Africa	Use of over the counter (OTC) simvastatin 10mg (a low dose statin) for the primary prevention of myocardial infarction (MI) compared to no drug-therapy. The base-case takes into account a 10-year CVD risk of 15% with an annual increase of 0.03% in CVD risk. A second scenario was evaluated in which the probabilities of fatal and non-fatal MI, independent of base-line CVD risk, were sourced from the Isfahan Cohort Study (ICS) population. In a third scenario, people aged 70 or more were given prescription statin (atorvastatin 10 mg) for primary prevention in both the IX and non-IX groups. Three different discounting scenarios were also considered.	ACER	Simvastatin, discount rate 0%, public prices, IX scenario	20.54
						Simvastatin, discount rate 0%, private prices, IX scenario	26.14
						Simvastatin with ICS mort/morb trans. prob. data, discount rate 0%, public prices, IX scenario	21.67
						Simvastatin with ICS mort/morb trans. prob. data, discount rate 0%, private prices, IX scenario	28.73
						Simvastatin with both control and IX group taking 10 mg atorvastatin at age 70+ , discount rate 0%, public prices, IX scenario	24.16
						Simvastatin with both control and IX group taking 10 mg atorvastatin at age 70+ , discount rate 0%, private prices, IX scenario	34.82
						Simvastatin, discount rate 3%, public prices, IX scenario	19.88
						Simvastatin, discount rate 3%, private prices, IX scenario	24.68
						Simvastatin with ICS mort/morb trans. prob. data, discount rate 3%, public prices, IX scenario	20.59
						Simvastatin with ICS mort/morb trans. prob. data, discount rate 3%, private prices, IX scenario	26.31
						Simvastatin with both control and IX group taking 10 mg atorvastatin at age 70+ , discount rate 3%, public prices, IX scenario	22.11
						Simvastatin with both control and IX group taking 10 mg atorvastatin at age 70+ , discount rate 3%, private prices, IX scenario	30.01
						Simvastatin, discount rate 7.2% for costs and 3% for effects, public prices, IX scenario	11.74
						Simvastatin, discount rate 7.2% for costs and 3% for effects, private prices, IX scenario	14.17
						Simvastatin with ICS mort/morb trans. prob. data, discount rate 7.2% for costs and 3% for effects, public prices, IX scenario	11.98
						Simvastatin with ICS mort/morb trans. prob. data, discount rate 7.2% for costs and 3% for effects, private prices, IX scenario	14.66
						Simvastatin with both control and IX group taking 10 mg atorvastatin at age 70+ , discount rate 7.2% for costs and 3% for effects, public prices, IX scenario	12.35
						Simvastatin with both control and IX group taking 10 mg atorvastatin at age 70+ , discount rate 7.2% for costs and 3% for effects, private prices, IX scenario	15.66
					ICER	Simvastatin, discount rate 0%, public prices, compared to no-drug scenario	1,009.86
						Simvastatin, discount rate 0%, private prices, compared to no-drug scenario	888.55
						Simvastatin with ICS mort/morb trans. prob. data, discount rate 0%, public prices, compared to no-drug scenario	795.68
						Simvastatin with ICS mort/morb trans. prob. data, discount rate 0%, private prices, compared to no-drug scenario	676.70
						Simvastatin with both control and IX group taking 10 mg atorvastatin at age 70+ , discount rate 0%, public prices, compared to no-drug scenario	932.87
						Simvastatin with both control and IX group taking 10 mg atorvastatin at age 70+ , discount rate 0%, private prices, compared to no-drug scenario	832.83
						Simvastatin, discount rate 3%, public prices, compared to no-drug scenario	1,483.85
						Simvastatin, discount rate 3%, private prices, compared to no-drug scenario	1,331.72
						Simvastatin with ICS mort/morb trans. prob. data, discount rate 3%, public prices, compared to no-drug scenario	1,222.36
						Simvastatin with ICS mort/morb trans. prob. data, discount rate 3%, private prices, compared to no-drug scenario	1,075.65
						Simvastatin with both control and IX group taking 10 mg atorvastatin at age 70+ , discount rate 3%, public prices, compared to no-drug scenario	1,478.72
						Simvastatin with both control and IX group taking 10 mg atorvastatin at age 70+ , discount rate 3%, private prices, compared to no-drug scenario	1,340.62
						Simvastatin, discount rate 7.2% for costs and 3% for effects, public prices, compared to no-drug scenario	932.41

						Simvastatin, discount rate 7.2% for costs and 3% for effects, private prices, compared to no-drug scenario	853.37
						Simvastatin with ICS mort/morb trans. prob. data, discount rate 7.2% for costs and 3% for effects, public prices, compared to no-drug scenario	782.93
						Simvastatin with ICS mort/morb trans. prob. data, discount rate 7.2% for costs and 3% for effects, private prices, compared to no-drug scenario	711.58
						Simvastatin with both control and IX group taking 10 mg atorvastatin at age 70+ , discount rate 7.2% for costs and 3% for effects, public prices, compared to no-drug scenario	1,043.33
						Simvastatin with both control and IX group taking 10 mg atorvastatin at age 70+ , discount rate 7.2% for costs and 3% for effects, private prices, compared to no-drug scenario	959.71
Borissov	Bulgaria	Upper middle	Europe and Central Asia	The standard of care scenario (control) was treating heterozygous familial hypercholesterolemia (HeFH) patients (with or without a history of CVD) with high-intensity statins (40-80 mg atorvastatin; 20–40 mg rosuvastatin). The intervention scenario inovlves treating HEFH patients with evolucumab, a PCSK9 inhibitor, in addition to the standard of care.	ACER	Status quo treatment (high-intensity statins) for patients with HeFH	1,048.90
						Evolocumab plus status quo treatment (high-intensity statins) for patients with HeFH	6,975.20
					ICER	Evolocumab plus status quo treatment (high-intensity statins) for patients with HeFH, compared to status quo treatment alone	77,952.01
Li	China	Upper middle	East Asia and Pacific	Newly diagnosed patients with type 2 diabetes were assigend to either a control group that did not receive statin therapy, or to a IX group that received statin therapy.	ACER	Control group (no statin therapy given to diabetic patients)	676.74
					ACER	Intervention group (statin therapy provided to diabetic patients)	776.72
					ICER	Intervention group (statin therapy provided to diabetic patients), compared to control group	24,957.19
Mould-Quevedo	Brazil and Colombia	Upper middle	Latin America and Caribbean	For the reference case analysis for primary prevention in Colombia, atorvastatin 10mg (A10) was compared with rosuvastatin 5mg (R5), and atorvastatin 20mg (A20) was compared with rosuvastatin 10mg (R10). In the Brazilian analysis, A20 was compared with both R10 and rosuvastatin 20mg (R20). For secondary prevention, the comparisons were atorvastatin 40mg (A40) versus R20 and atorvastatin 80mg (A80) versus rosuvastatin 40 mg (R40 for Colombia and A40 versus R40 for Brazil.	ACER	Colombia, primary prevention, atorvastatin 10 mg	293.98
						Colombia, primary prevention, rosuvastatin 5 mg	579.20
						Colombia, primary prevention, atorvastatin 20 mg	412.70
						Colombia, primary prevention, rosuvastatin 10 mg	658.52
						Colombia, secondary prevention, atorvastatin 40 mg	1,064.75
						Colombia, secondary prevention, rosuvastatin 20 mg	1,362.73
						Colombia, secondary prevention, atorvastatin 80 mg	1,174.29
						Colombia, secondary prevention, rosuvastatin 40 mg	1,554.04
						Brazil, primary prevention, atorvastatin 20 mg	203.27
						Brazil, primary prevention, rosuvastatin 10 mg	204.75
						Brazil, primary prevention, rosuvastatin 20 mg	317.60
						Brazil, secondary prevention, atorvastatin 40 mg	401.92
						Brazil, secondary prevention, rosuvastatin 40 mg	454.79
					ICER	Colombia, primary prevention, rosuvastatin 5 mg, compared to atorvastatin 10 mg	762,765.24
						Colombia, primary prevention, rosuvastatin 10 mg, compared to atorvastatin 20 mg	658,184.77
						Colombia, secondary prevention, rosuvastatin 20 mg, compared to atorvastatin 40 mg	209,999.43
						Colombia, secondary prevention, rosuvastatin 40 mg, compared to atorvastatin 80 mg	269,086.02
						Brazil, primary prevention, rosuvastatin 10 mg, compared to atorvastatin 20 mg	4,098.86
						Brazil, primary prevention, rosuvastatin 20 mg, compared to atorvastatin 20 mg	127,746.10
						Brazil, secondary prevention, rosuvastatin 40 mg, compared to atorvastatin 40 mg	18,506.67

Supplementary Table 8: Cost effectiveness per 1% reduction in LDL-C, 2019 USD

First Author	Country	WB Income Level	Region	IX Description	ACER or ICER	Intervention Subgroup	Cost in 2019 USD
Briseno	Mexico	Upper middle	Latin America and Caribbean	Patients with or without established CVD received drug therapy with either oral rosuvastatin 10 mg/day or oral fixed dose combination ezetimibe/simvastatin 10/20 mg/day once daily.	ACER	Rosuvastatin	2.56
						Fixed dose combination ezetimibe/simvastatin	5.19
Sansana-yudh	Thailand	Upper middle	South Asia	Treatment with Pitavastatin 1mg/d or Atorvastatin 10mg/d among patients older than 18 years of age with hypercholesterolemia who had an indication for statin therapy according to the NCEP-ATP III guidelines.	ACER	Pitavastatin 1mg/d	0.91
						Atorvastatin 10mg/d	1.85
